# De Novo Design and Directed Evolution Refinement of Mirror‐Image Protein Binders Targeting Interleukin‐4

**DOI:** 10.1002/advs.202515425

**Published:** 2026-03-31

**Authors:** Liqing Xu, Yuxiang Ren, Tongyue Wang, Hongyong Li, Yue Yao, Lei Liu, Ke Sun, Peilong Lu

**Affiliations:** ^1^ College of Pharmaceutical Sciences Zhejiang University Hangzhou Zhejiang China; ^2^ Westlake Laboratory of Life Sciences and Biomedicine Hangzhou Zhejiang China; ^3^ Key Laboratory of Structural Biology of Zhejiang Province School of Life Sciences Westlake University Hangzhou Zhejiang China; ^4^ Institute of Biology Westlake Institute for Advanced Study Hangzhou Zhejiang China; ^5^ Tsinghua‐Peking Joint Center for Life Sciences Ministry of Education Key Laboratory of Bioorganic Phosphorus Chemistry and Chemical Biology Center for Synthetic and Systems Biology Department of Chemistry Tsinghua University Beijing China

**Keywords:** D‐protein inhibitor, IL‐4, mirror‐image protein, protein binder, protein design

## Abstract

Human interleukin‐4 (IL‐4) is a critical therapeutic target for allergic diseases and cancer, yet current biologics face stability and specificity limitations. We report a novel strategy combining de novo computational design with directed evolution to engineer a D‐protein inhibitor targeting IL‐4. Unlike stochastic screening, our approach enables epitope‐specific design against the mirror‐image D‐IL‐4 structure. Crucially, we integrated WALTZ‐guided aggregation prediction into the evolution cycle to simultaneously optimize both binding affinity and solution behavior. The resulting D‐protein, D‐18252‐evo, binds native IL‐4 with nanomolar affinity (∼87 nM) and effectively blocks receptor engagement. Functional assays confirm potent inhibition of IL‐4‐induced STAT6 phosphorylation and cell proliferation. Furthermore, D‐18252‐evo exhibits exceptional biophysical properties, including high thermal stability and resistance to proteolytic degradation. This work establishes a scalable framework for generating robust mirror‐image therapeutics, positioning D‐proteins as a promising next‐generation platform for treating cytokine‐driven disorders with enhanced stability and targeted efficacy.

## Graphical abstract

This study presents the de novo design and directed evolution of a mirror‐image D‐protein inhibitor targeting human interleukin‐4 (IL‐4). The engineered molecule exhibits nanomolar binding affinity for IL‐4 and effectively inhibits IL‐4–mediated signaling. Its remarkable resistance to proteolytic degradation and thermal denaturation underscores the therapeutic potential of abiotic D‐proteins in the treatment of immune‐mediated diseases.

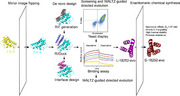

## 1 Introduction

Human interleukin‐4 (IL‐4) is a key regulatory cytokine involved in Th2‐type immune responses [1]. It contributes to the pathogenesis of allergic disorders, including atopic dermatitis and asthma, primarily through activation of the STAT6 signaling pathway [2]. Additionally, IL‐4 plays a role in tumor immune evasion by modulating the tumor microenvironment [3]. This cytokine is predominantly secreted by mast cells, Th2 lymphocytes, eosinophils, and basophils [4]. IL‐4 promotes immunoglobulin E (IgE) synthesis in B lymphocytes [5], thereby enhancing mast cell activation and driving allergic inflammation. At the molecular level, IL‐4 initially binds to the extracellular domain of the IL‐4 receptor α subunit (IL‐4Rα) [6, 7], which then recruits either the common γ‐chain (γc) to form the type I receptor complex or the interleukin‐13 receptor subunit alpha‐1 (IL‐13Rα1) to form the type II receptor complex [8, 9, 10]. This receptor engagement leads to activation of the STAT6 transcription factor, which, upon phosphorylation, translocates to the nucleus to regulate gene expression—a mechanism implicated in atopic dermatitis, asthma, and allergic rhinitis [11, 12, 13, 14]. Recent studies indicate that IL‐4 expression within the tumor microenvironment can confer resistance to anti‐PD‐1 therapy in ovarian cancer [15], highlighting IL‐4 as a promising therapeutic target in both inflammatory diseases and oncology [16, 17, 18].

Current therapeutic strategies targeting IL‐4 signaling predominantly focus on monoclonal antibodies, most of which are directed against IL‐4Rα [19]. Dupilumab, an IL‐4Rα antagonist, has achieved clinical success by inhibiting the shared IL‐4Rα subunit essential for both IL‐4 and IL‐13 signaling pathways and is now approved for the treatment of moderate to severe atopic dermatitis and asthma [20, 21]. In contrast, pascolizumab [22]—a humanized antibody that directly neutralizes IL‐4—showed limited efficacy in clinical trials and was subsequently discontinued [23]. Additionally, small‐molecule inhibitors designed to disrupt the IL‐4/IL‐4Rα interaction have been investigated, but their development has been impeded by low binding affinity and limited cellular potency [24]. These challenges highlight the limitations of current IL‐4‐targeted therapies and underscore the need for novel and more effective therapeutic approaches.

Among promising alternatives, D‐proteins—composed of D‐amino acids and the achiral amino acid glycine [25]—represent a compelling strategy for developing protein therapeutics. Compared to conventional L‐proteins, D‐proteins offer distinct biological advantages, including reduced immunogenicity, enhanced resistance to proteolytic degradation, and extended in vivo half‐life [26]. D‐protein binders can be developed through iterative mirror‐image phage display of small protein scaffold libraries against chemically synthesized D‐protein targets [27]. Foundational work by Kent and colleagues established that this strategy could be extended from D‐peptides to compact D‐protein binders, beginning with a GB1‐derived VEGF‐A antagonist, and subsequent engineering further improved stability, affinity, and in vivo efficacy [28, 29, 30]. Beyond mirror‐image phage display, the scope of mirror‐image binder discovery has expanded to encompass high‐diversity platforms enabled by chemical synthesis. Notably, mirror‐image TRAP display has been utilized to generate mirror‐image monobodies targeting MCP‐1 [31]. Concurrently, mirror‐image monobodies targeting oncogenic proteins, such as the BCR::ABL1 SH2 domain, have been developed via phage display followed by yeast‐display‐based selection against a synthetic D‐target [32]. Collectively, these developments build upon and were enabled by foundational advances in chemical protein synthesis, such as native chemical ligation (NCL), and structure‐determination strategies, including racemic and quasi‐racemic protein crystallography, pioneered by Kent and colleagues [28, 29, 30, 32, 33, 34, 35, 36, 37]. However, these methodologies primarily depend on stochastic library screening, which frequently provides limited control over the specific binding epitope.

To address the limitations of current screening paradigms, we introduce a rational, epitope‐driven design framework that integrates computational de novo design with directed evolution. Unlike stochastic approaches, our method utilizes a high‐resolution structural model to target specific functional interfaces. Recent advances include the development of high‐affinity D‐proteins targeting complex biomolecules such as interleukin‐6 [38].

**FIGURE 1:**
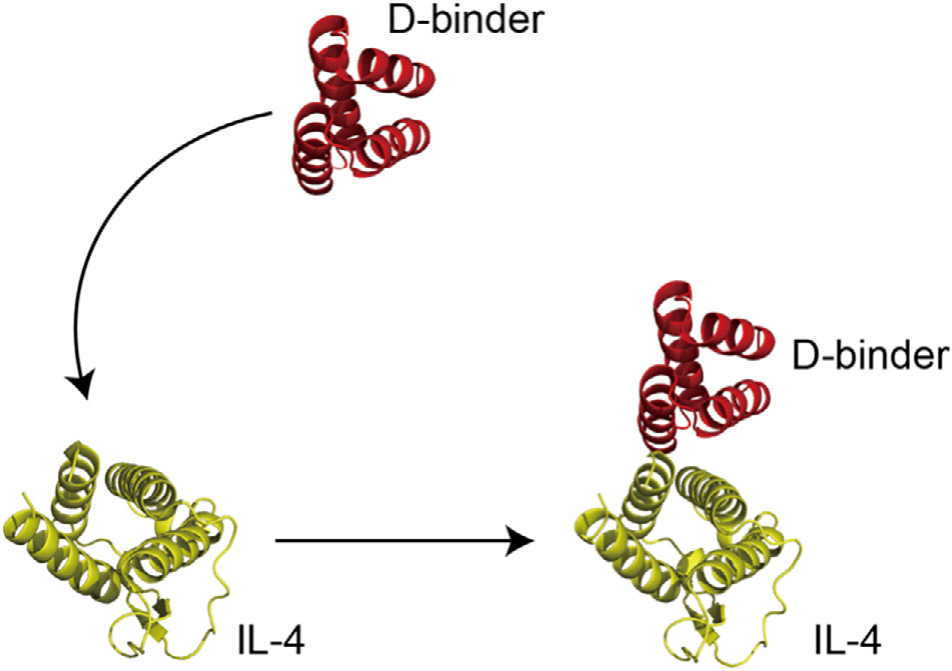
Cartoon representation of the inhibition of IL‐4 binding to the IL‐4Rα by a D‐protein binder. The D‐protein binder (red) occupies the IL‐4Rα binding site on human IL‐4 (yellow), thereby preventing receptor engagement and inhibiting downstream IL‐4 signaling.

In this study, we further incorporated WALTZ [39], a computational predictor of amyloidogenic sequences, as an in silico filter during the evolution cycle. This strategy allows for the simultaneous optimization of binding affinity and solution behavior, deprioritizing aggregation‐prone variants early in the process. We applied this integrated workflow to develop a potent D‐protein inhibitor of human IL‐4 (Figure 1). By designing L‐protein binders against a computationally mirrored D‐IL‐4 structure (IL‐4–IL‐4Rα–IL‐13Rα1; PDB ID: 3BPN) [7] and refining them through directed evolution, we generated a candidate that was chemically synthesized as its D‐enantiomer. The resulting D‐protein binder exhibits nanomolar affinity for native IL‐4, effectively disrupts receptor engagement, and suppresses downstream STAT6 signaling. With its exceptional thermal stability and protease resistance, this engineered D‐protein establishes a robust platform for next‐generation biotherapeutics targeting IL‐4‐driven diseases.

## 2 Results

### 2.1 The Mirror‐Image Design Approach for Targeting Human Interleukin‐4

**FIGURE 2:**
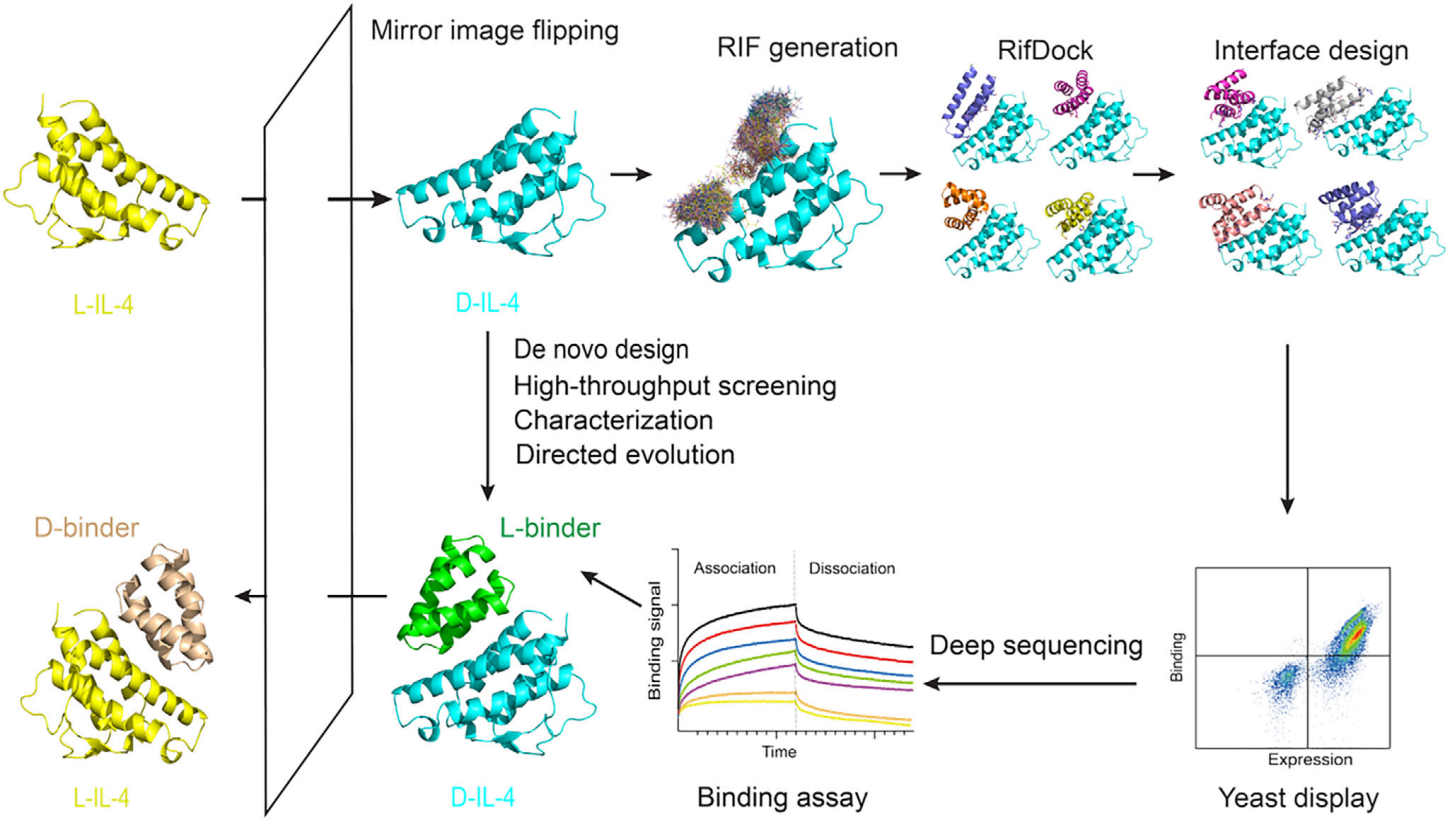
Design and characterization of D‐protein binders targeting human IL‐4. The upper panel depicts the computational design workflow. The structure of human L‐IL‐4 (yellow) was mirrored to generate D‐IL‐4 (cyan). A structural epitope on D‐IL‐4, corresponding to the native binding interface between human IL‐4 and IL‐4Rα, was selected for binder design. This epitope comprises residues I29, T30, Q32, E33, K36, T37, S40, E43, Q44, H98, R99, K101, Q102, R105, F106, R109, R112, N113, and L117. RIFs were generated against this epitope using RifGen, followed by docking of mini‐protein scaffolds with RifDock. The heterochiral interface was subsequently optimized through computational design using Rosetta. The lower panel outlines the experimental validation workflow. High‐affinity L‐protein binders (green) against D‐IL‐4 (cyan) were identified by sequencing a yeast surface display library [42] after fluorescence‐activated cell sorting. The corresponding D‐protein binders (wheat), chemically synthesized using D‐amino acids and sharing identical sequences with their L‐protein counterparts, were shown to bind human L‐IL‐4 (yellow).

We employed an integrated computational and experimental approach [38] to develop high‐affinity D‐protein binders targeting IL‐4 (Figure 2). First, we generated the mirror‐image D‐IL‐4 molecule by computationally inverting the chirality of all amino acids in the native L‐IL‐4 structure (hereafter referred to as L‐IL‐4). For D‐IL‐4, the design focused on a structural epitope corresponding to the IL‐4Rα‐binding region of L‐IL‐4 [7].

Using the RifGen module, we constructed a rotamer interaction field (RIF) based on the D‐IL‐4 epitope. The RifDock module was subsequently used to screen an L‐protein scaffold library containing five distinct topologies against the RIF, aiming to identify potential binders to D‐IL‐4. The L‐protein scaffolds used as starting points are small, de novo designed miniproteins originally developed by Cao et al. [40]. These small proteins were generated using fragment assembly, piecewise fragment assembly, and helical extension, allowing for diverse topologies and well‐packed hydrophobic cores. Their structural stability was experimentally validated in the original study via a high‐throughput proteolysis‐based assay, confirming their robustness and suitability as reliable scaffold templates. Following initial interface design, we applied MotifGraft [41] to extract key interaction motifs and graft them onto diverse mini‐protein scaffolds, thereby enabling the exploration of additional heterochiral binding geometries.

Potential L‐protein binders were identified through multiple rounds of high‐throughput screening using yeast surface display, with enriched sequences detected by next‐generation sequencing. The top candidates were expressed in *Escherichia coli*, purified and evaluated for binding affinity to chemically synthesized D‐IL‐4 using BLI. Directed evolution was then applied to improve the binding affinity and functional characteristics of the lead variants.

Finally, the D‐protein binder corresponding to the highest‐affinity L‐protein sequence was synthesized chemically and characterized. Functional validation encompassed measurements of binding affinity to human L‐IL‐4, resistance to proteolytic degradation, and inhibition of IL‐4 signaling.

### 2.2 L‐ and D‐Protein Binders: Design, Purification, and Binding Analysis

**FIGURE 3:**
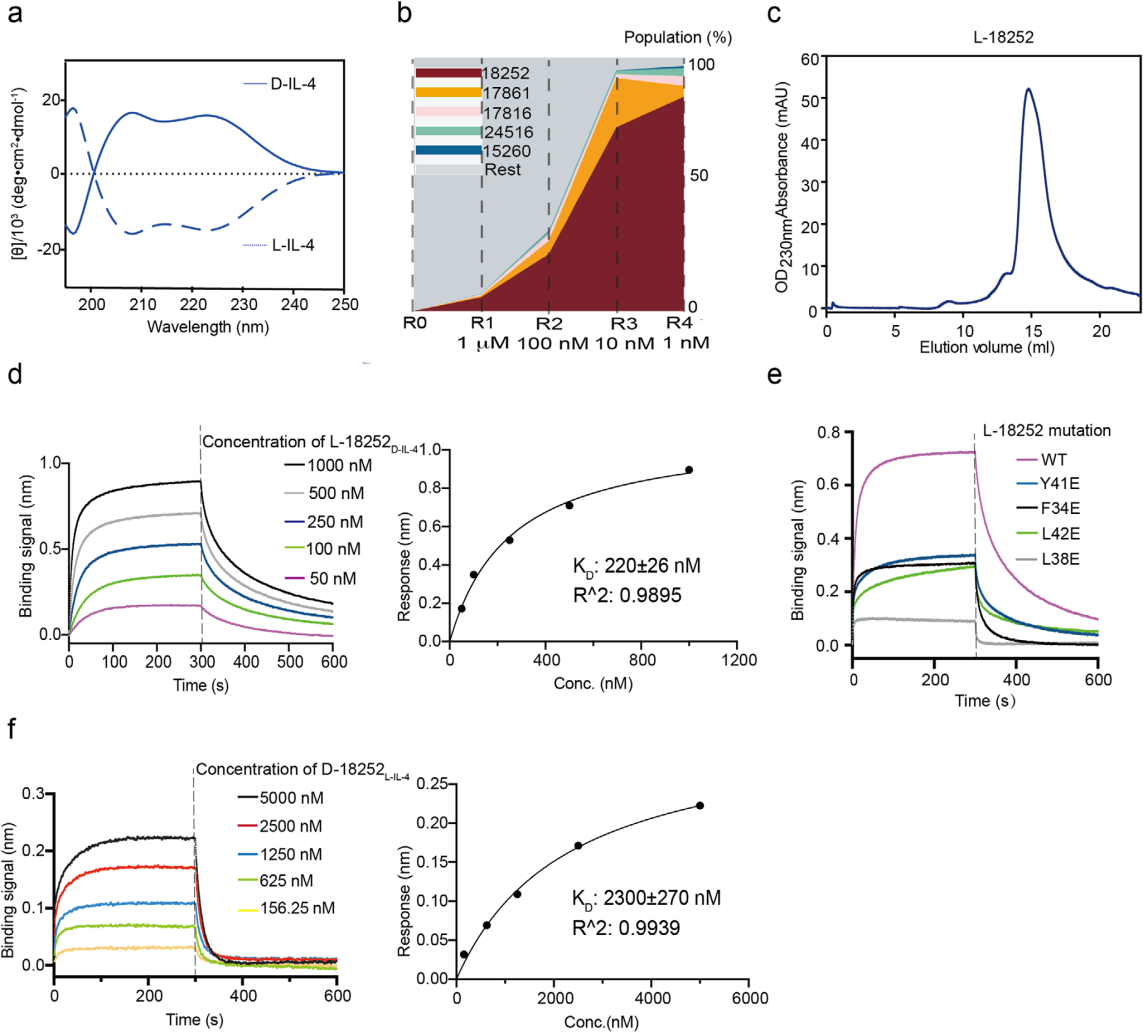
Design, characterization, and binding analysis of L‐ and D‐IL‐4 protein binders. (a) CD spectra of D‐IL‐4 (solid line) and L‐IL‐4 (dashed line) measured in a 0.5 mm path‐length quartz cuvette with protein concentration at 0.2 mg/mL. Each trace represents the average of two consecutive scans (technical replicates, *n* = 2). (b) High‐throughput screening results showing four rounds of sorting at progressively decreasing target concentrations, with enrichment levels quantified by NGS. (c) SEC of purified L‐18252 on a Superdex 75 Increase column. (d) Binding affinity of L‐18252 to D‐IL‐4 determined by BLI using steady‐state analysis with a concentration series. A 1:1 binding model in Octet Data Analysis software yielded K_D_ = 220 ± 26 nm (R^2^ = 0.9895). Complete fitting parameters are provided in Table S1. (e) BLI analysis of L‐18252 interface mutants. Each mutant was tested at a 1 µM analyte concentration against immobilized D‐IL‐4; representative sensorgrams are displayed. (f) The binding of D‐18252 to L‐IL‐4 was quantified by steady‐state BLI analysis and fitted to a 1:1 binding model, yielding a dissociation constant of K_D_ = 2300 ± 270 nm (R^2^ = 0.9939). Complete fitting parameters are provided in Table S1.

We chemically synthesized the D‐enantiomer of human interleukin‐4 (D‐IL‐4, residues 25–153) bearing an N‐terminal biotin tag (Figure S1) via hydrazide‐based NCL [43, 44, 45, 46, 47]. The crude D‐IL‐4 was solubilized in 6 M guanidinium chloride (Gd·Cl) and folded via dilution into a glutathione‐based redox buffer (GSH/GSSG), followed by dialysis against PBS to remove the denaturant. The folded protein was subsequently purified by size‐exclusion chromatography (SEC) (Figure S2). High‐resolution mass spectrometry (HRMS) confirmed an intact mass corresponding to the fully oxidized form, consistent with the formation of three disulfide bonds (Figure S3). CD spectroscopy revealed a spectrum that is the mirror image of recombinant L‐IL‐4 produced in *Escherichia coli* (Figure 3a), indicating that D‐IL‐4 adopts the expected enantiomeric secondary structure. Together, these results support that synthetic D‐IL‐4 is correctly folded and suitable for downstream selection and binding studies.

From our design pipeline, we selected 7,888 L‐protein binders, each 50–65 amino acids in length, that were predicted to be at least locally optimal based on computational design criteria. These binders were sampled from an estimated sequence space of approximately **10^20^**, derived from ∼10 000 protein backbones **×** 10^16^ possible combinations of interface residues. The genes encoding these designed binders were synthesized into an oligonucleotide library and expressed on the surface of yeast cells. We conducted four rounds of fluorescence‐activated cell sorting (FACS) using the yeast‐displayed library, progressively reducing the concentration of folded D‐IL‐4 to 1 µm, 100 nm, 10 nm, and 1 nm. After each selection round, enriched binder sequences were analyzed by next‐generation sequencing (NGS), which identified L‐18252 as the most highly enriched clone (Figure 3b).

L‐18252 was expressed in *Escherichia coli* and purified using Ni^2^^+^‐affinity chromatography (IMAC). SDS‐PAGE analysis of the affinity‐purified eluate showed significant protein aggregation and substantial co‐elution of host cell proteins (Figure S4a). The L‐18252 protein was further purified using SEC and analyzed by SDS‐PAGE (Figure S4b). The SEC profile showed a broad, heterogeneous peak eluting between 8 and 11 mL in addition to the target protein. Quantitative integration revealed that aggregate‐ and host‐protein complexes accounted for more than 50% of the total chromatographic area. These findings indicate that L‐18252 has suboptimal solution properties and a pronounced tendency to aggregate, consistent with intrinsic structural limitations. To achieve high sample purity and improved biophysical properties, the primary SEC peak was isolated and subjected to a second round of SEC (Figure 3c), with the final product confirmed by SDS‐PAGE (Figure S6a). Similar purification procedures were applied to the evolved variants and L‐binder mutants (Figures S5 and S6b–f). Despite these characteristics, L‐18252 bound to D‐IL‐4 with sub‐micromolar affinity, yielding an equilibrium dissociation constant (K_D_) of 220 ± 26 nm (Figure 3d). Four interfacial residues in L‐18252—Phe34, Leu38, Tyr41, and Leu42—were selected for mutagenesis studies, with each residue individually substituted by glutamic acid. All protein variants were successfully expressed and purified to homogeneity, as confirmed by SEC and SDS‐PAGE (Figures S5 and S6c–f). Binding interactions were assessed using BLI with streptavidin biosensors loaded with biotinylated D‐IL‐4, and each mutant protein was tested at a consistent concentration of 1 µM. All four mutants displayed significantly reduced binding signals relative to the wild‐type L‐18252 (Figure 3e), demonstrating that these residues are essential for interface formation and play a substantial role in binding affinity.

Then, we chemically synthesized the mirror‐image enantiomer of L‐18252, designated D‐18252 (Figure S7), and purified the folded protein to assess its binding to L‐IL‐4. BLI binding assays revealed that D‐18252 binds L‐IL‐4 with a K_D_ of 2300 ± 270 nm (Figure 3f), reflecting a substantial reduction in affinity compared to the interaction between L‐18252 and D‐IL‐4 (K_D_ = 220 ± 26 nm). This marked decrease in binding affinity upon chirality inversion indicates that the suboptimal biophysical properties of L‐18252, such as its propensity to aggregate with host proteins as previously observed, likely impair the strength of the interaction.

### 2.3 Computational Modeling and Interface Analysis of the L‐18252–D‐IL‐4 Complex

**FIGURE 4:**
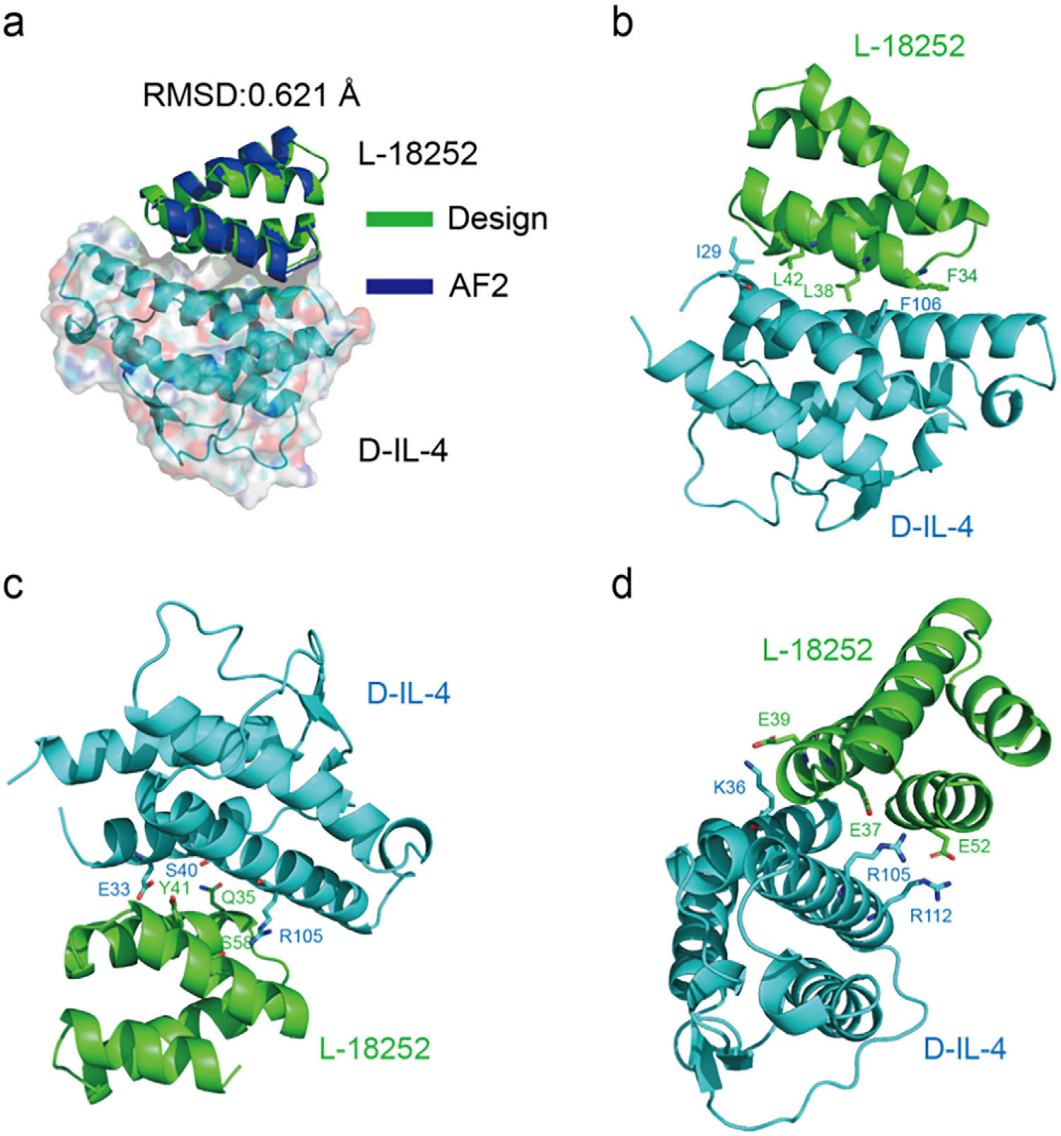
Computational Modeling of the L‐18252–D‐IL‐4 Interaction Interface. (a) Structural prediction of L‐18252 using AlphaFold2 (blue) closely aligns with the original design model (green), displayed in complex with D‐IL‐4 in both surface and cartoon representations. (b) Key hydrophobic interactions at the designed interface between L‐18252 and D‐IL‐4 in the computational model. (c) Polar interactions within the designed binding interface of the L‐18252/D‐IL‐4 complex in the computational model. (d) Electrostatic interactions in the modeled L‐18252/D‐IL‐4 complex in the computational model. Interacting residues are displayed as stick representations.

The structure of L‐18252 was predicted using AlphaFold2 and closely matched the designed model, with a root mean square deviation (RMSD) of less than 1 Å for Cα atoms (Figure 4a), supporting that L‐18252 adopts the intended folded conformation. Importantly, AlphaFold2 was not utilized during the design process, which relied exclusively on Rosetta‐driven heterochiral interface design and energy‐based scoring. The close agreement between the predicted and designed structures offers an orthogonal computational cross‐check of the intended fold of L‐18252, underscoring the accuracy and robustness of our de novo design approach. We further examined the Rosetta‐generated model of the L‐18252 and D‐IL‐4 complex, identifying several interfacial residues predicted to mediate distinct types of molecular interactions. Notably, Phe34, Leu38, and Leu42 of L‐18252 form extensive hydrophobic interactions with Ile29 and Phe106 of D‐IL‐4, contributing to a tightly packed nonpolar core at the binding interface (Figure 4b). Additional stabilization is predicted by multiple hydrogen bonds (Figure 4c), including those between Tyr41 (L‐18252) and Glu33 (D‐IL‐4), Gln35 (L‐18252) and Ser40 (D‐IL‐4), and Ser58 (L‐18252) and Arg105 (D‐IL‐4). The model also reveals three well‐defined salt bridges formed between Glu37, Glu39, and Glu52 of L‐18252 and Arg105, Lys36, and Arg112 of D‐IL‐4, respectively (Figure 4d). Collectively, these interactions offer a structural explanation for the observed affinity and specificity at the protein–protein interface.

### 2.4 Directed Evolution of L‐18252

To enhance both binding affinity and protein solution behavior, we constructed a site‐saturation mutagenesis (SSM) library, followed by combinatorial mutagenesis and error‐prone PCR mutagenesis libraries. Directed evolution [48, 49] has been widely demonstrated to improve protein biophysical properties by introducing mutations that reduce surface hydrophobicity and eliminate aggregation‐prone regions. To identify potential amyloidogenic sequences, we employed the WALTZ [39] prediction tool, a sequence‐based predictor that identifies short hexapeptide segments with a high propensity for forming ordered amyloid‐like assemblies, indicative of local aggregation or amyloidogenic potential. In contrast to earlier prediction methods, WALTZ combines amino acid sequence scoring with physicochemical property analysis to identify short segments with a high propensity to form ordered amyloid fibrils. By integrating high‐throughput sequencing data from our mutagenesis libraries with WALTZ predictions, we identified and selected three variants with reduced amyloidogenic potential for experimental validation. These engineered mutants exhibited markedly reduced aggregation with host proteins and improved purity, consistent with enhanced biophysical properties, as supported by SEC and SDS‐PAGE analysis. Among the variants, L‐18252‐evo displayed the most favorable behavior during purification. Under identical conditions to the parental variant L‐18252, L‐18252‐evo showed reduced aggregation with host proteins, as evidenced by both SEC and SDS‐PAGE (Figure S4), indicating higher purity and improved solution behavior. Comparative SDS‐PAGE of the Ni^2^^+^‐affinity eluates (Figure S4a) corroborated this observation: L‐18252‐evo co‐eluted with markedly fewer host‐cell contaminants, whereas the parental L‐18252 exhibited pronounced aggregation and contamination.

By contrast, the SEC profiles of L‐18252‐evo featured a sharp, symmetric principal peak with only minor contaminant contributions (Figure S4c). A reliable purification yield was determined by quantifying the high‐purity main fraction from the SEC main peak, yielding 1.22 mg/L for L‐18252 and 1.05 mg/L for L‐18252‐evo. This result supports that directed evolution successfully minimized aggregation and non‐specific binding to host‐derived proteins, thereby enhancing the homogeneity and purity of the binder while maintaining a comparable overall yield.

**FIGURE 5:**
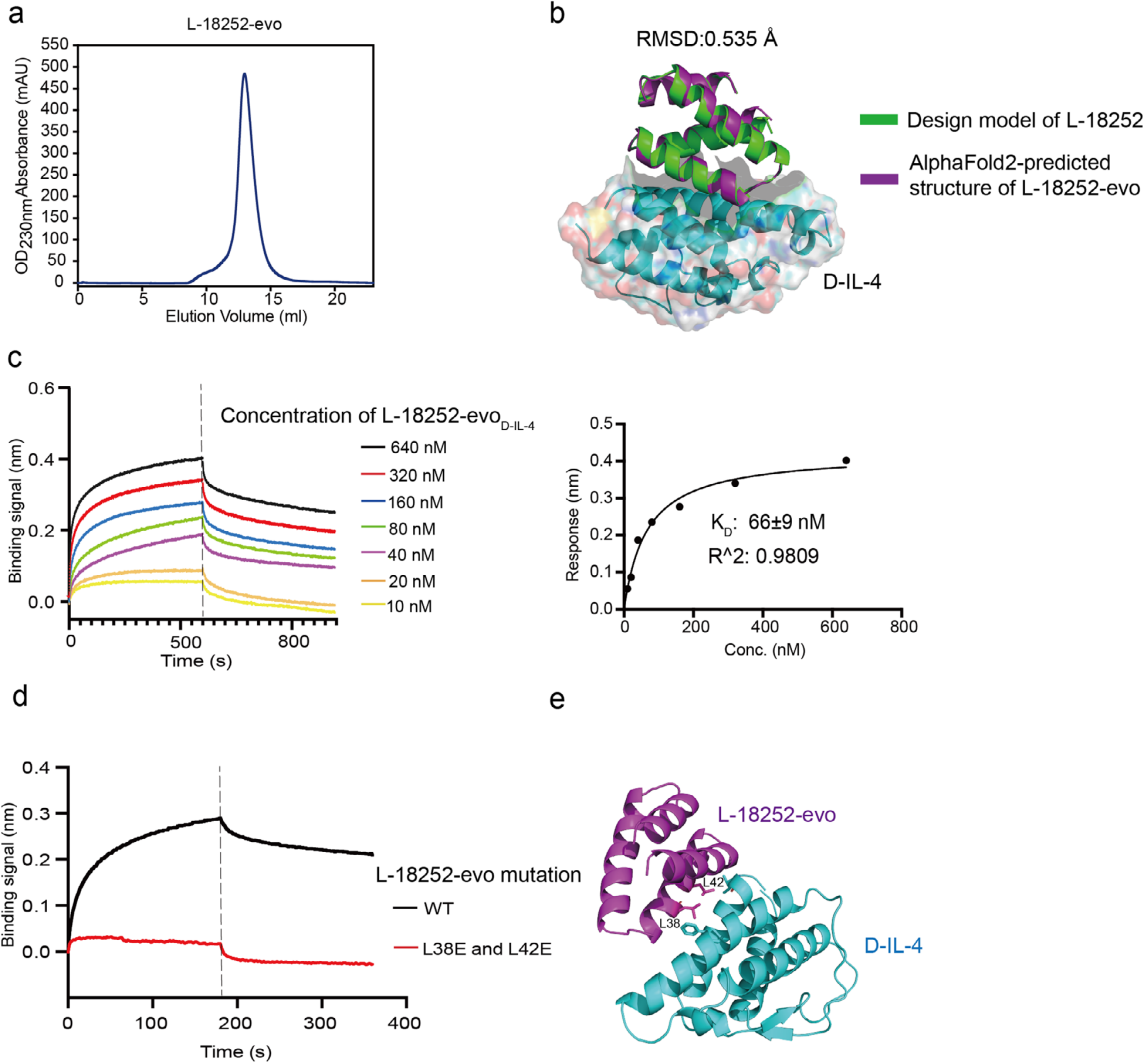
Evaluation of evolved L‐18252‐evo. (a) SEC profile of L‐18252‐evo on a Superdex 75 column. (b) Post hoc AlphaFold2‐predicted structure of L‐18252‐evo (purple) closely aligns with the computational design model (green), with an overall Cα RMSD < 1 Å. Surface and cartoon representations of D‐IL‐4 are shown for comparison. (c) Binding interaction between L‐18252‐evo and D‐IL‐4 was analyzed by steady‐state BLI and fitted to a 1:1 binding model using Octet Data Analysis, yielding a K_D_ of 66 ± 9 nm and an R^2^ value of 0.9809. Steady‐state fitting results, including K_D_ values and associated fit statistics, are provided in Table S1. (d) The contribution of individual mutations in L‐18252‐evo to D‐IL‐4 binding was assessed by BLI. (e) Key interfacial residues in the design model are shown as stick representations.

To ensure consistency with the original construct, the main peak fraction was collected and re‐analyzed by SEC (Figure 5a and Figure S6b) for downstream characterization. L‐18252‐evo contains eleven mutations relative to L‐18252: T9A, R10Y, V11E, L21I, N31T, Q35H, E39D, L40I, L43M, R56D, and R63N (Figure S8). Of these, Q35H and E39D are located at the binding interface, while the remaining substitutions are situated on solvent‐exposed surfaces. This spatial distribution suggests that the improved solution behavior primarily arises from favorable modifications in surface chemical properties that mitigate co‐aggregation with host‐derived proteins. To evaluate whether the accumulated mutations disrupt the intended protein fold, we independently predicted the structure of L‐18252‐evo using AlphaFold2. The predicted model (Figure 5b, purple) exhibits strong agreement with the designed structure (green), with a Cα RMSD of less than 1 Å for aligned residues. This high degree of structural similarity suggests that the sequence of L‐18252‐evo is compatible with the intended fold. Biolayer interferometry measurements demonstrated a double‐digit nanomolar binding affinity between L‐18252‐evo and D‐IL‐4 (K_D_ = 66 ± 9 nm), as determined by steady‐state analysis (Figure 5c). Furthermore, mutating key interfacial residues Leu38 and Leu42 to glutamic acid in L‐18252‐evo markedly impaired its binding to D‐IL‐4 (Figure 5d,e). Together, these results strongly support the conclusion that L‐18252‐evo binds D‐IL‐4 through the designed interaction interface.

### 2.5 Characterization of D‐18252‐evo

**FIGURE 6:**
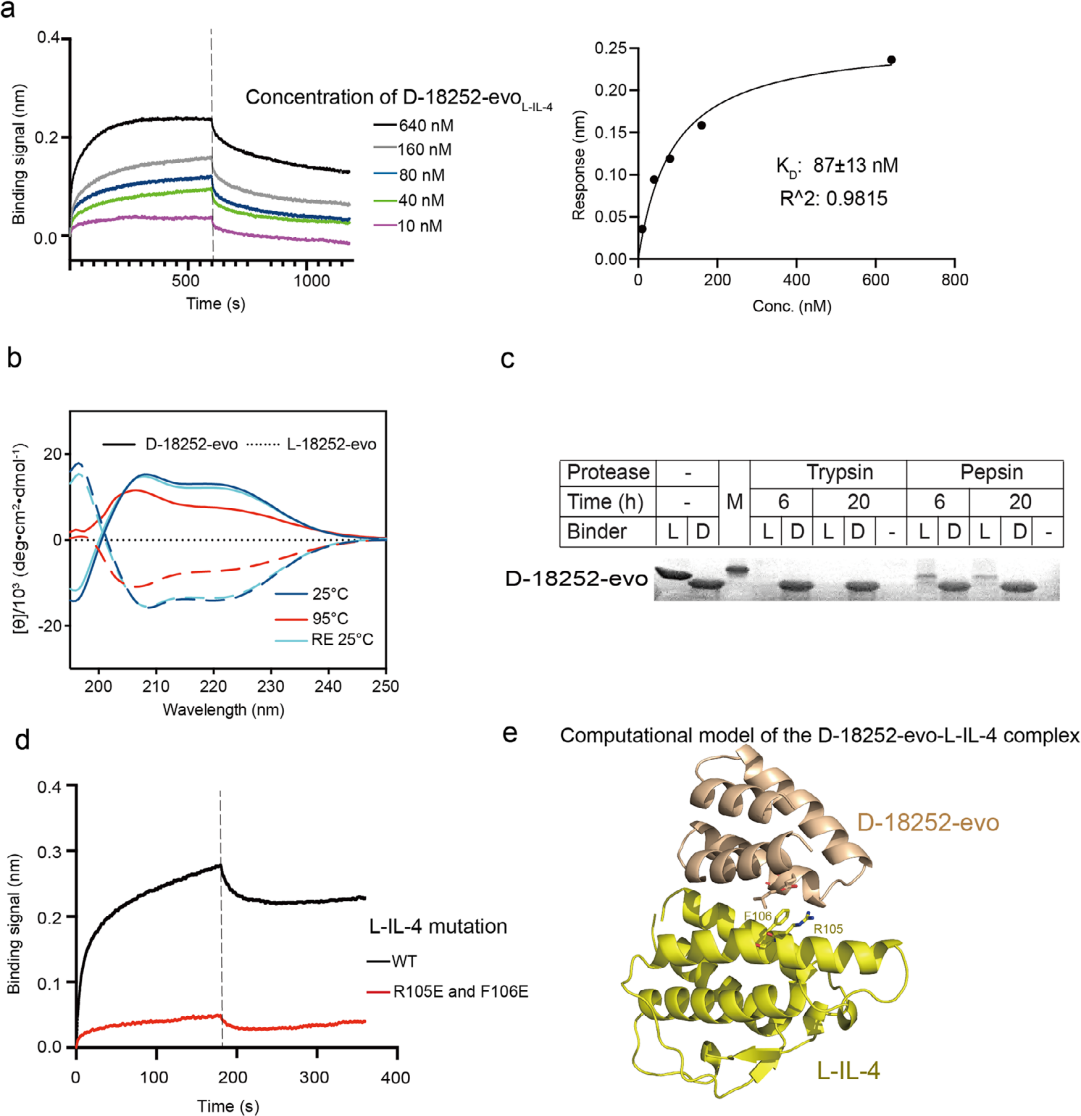
Validation of D‐18252‐evo. (a) Steady‐state responses were fit in Octet Data Analysis using a 1:1 binding model, yielding K_D_ = 87 ± 13 nm (best‐fit±fitting error; R^2^ = 0.9815). Parameters derived from steady‐state analysis are summarized in Table S1. (b) Far‐UV circular dichroism spectra of D‐18252‐evo (solid line) and L‐18252‐evo (dashed line) recorded at 25°C, 95°C, and after cooling to 25°C. Each spectrum represents the average of two consecutive scans (technical replicates; *n* = 2).  (c) Protease resistance of D‐18252‐evo and L‐18252‐evo was evaluated by SDS‐PAGE following incubation with trypsin or pepsin for 6 or 20 h under conditions described in the Methods. M denotes the molecular weight marker. A representative gel from two independent experiments (*n* = 2) is shown.  (d) L‐IL‐4 interface mutations selected on the basis of computational modeling and their binding affinities to D‐18252‐evo, as determined by BLI. (e) Key interfacial residues in the D‐18252‐evo–L‐IL‐4 computational model are shown as sticks.

Based on these findings, we chemically synthesized the mirror‐image binder protein D‐18252‐evo (Figure S9). The protein was folded under standard conditions—initial solubilization in 6 M Gd·Cl followed by dialysis into denaturant‐free buffer—a well‐established protocol for obtaining correctly folded proteins [38, 51]. Given that D‐18252‐evo is α‐helical and lacks disulfide bonds, this folding procedure is typically sufficient for α‐helical proteins that lack disulfide bonds and was validated in this study by CD and functional binding assays. BLI binding assays demonstrated that D‐18252‐evo binds L‐IL‐4 with an affinity of 87 ± 13 nm (Figure 6a), comparable to that of L‐18252‐evo (66 ± 9 nm), indicating similar binding characteristics. CD spectroscopy confirmed that D‐18252‐evo adopts a well‐defined α‐helical conformation and exhibits a spectrum inverted relative to its L‐enantiomer, L‐18252‐evo, as anticipated for enantiomeric proteins. Moreover, D‐18252‐evo exhibited high thermal stability in thermal denaturation experiments (Figure 6b). In protease resistance assays, D‐18252‐evo showed markedly enhanced stability compared to its L‐enantiomer: while L‐18252‐evo was nearly completely degraded by trypsin or pepsin within 6 h, D‐18252‐evo remained intact after 20 h of incubation (Figure 6c). Collectively, these results support that D‐18252‐evo is properly folded and functional, maintaining its designed binding affinity while demonstrating enhanced stability.

To investigate the binding mode of D‐18252‐evo to L‐IL‐4, we generated mutations in L‐IL‐4. Substitution of both Arg105 and Phe106 with glutamic acid substantially reduced binding to D‐18252‐evo (Figure 6d,e). These results are consistent with the binding mode predicted by the design model.

Additionally, SEC analysis supported that D‐18252‐evo forms a stable complex with L‐IL‐4, eluting as a single, well‐defined peak (Figure S10a). This complex formation was further validated by SDS‐PAGE with Coomassie staining (Figure S10b). Collectively, these results confirm the successful development of a de novo‐designed protein binder, D‐18252‐evo, exhibiting nanomolar affinity for L‐IL‐4 after refinement by directed evolution. These findings highlight the therapeutic potential of this D‐protein for the treatment of IL‐4‐mediated diseases.

### 2.6 Functional Validation of D‐18252‐evo in Cellular Assays

**FIGURE 7:**
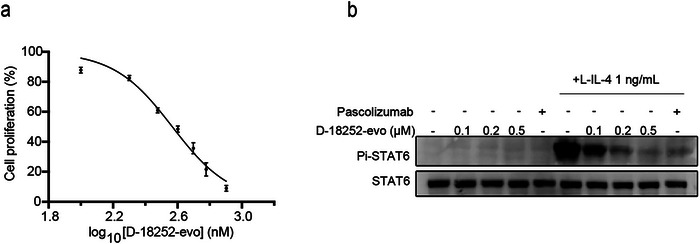
Functional validation of D‐18252‐evo. (a) Dose–response curve of TF‐1 cell proliferation following treatment with increasing concentrations of D‐18252‐evo in the presence of 1 ng/mL L‐IL‐4. Data points show the mean±SD of duplicate wells (technical replicates). The experiment was independently repeated twice (*n* = 2 independent experiments), yielding similar IC_50_ values. The curve was fitted using GraphPad Prism 10 with a four‐parameter logistic model (log(inhibitor) versus normalized response), yielding an IC_50_ of 369.9 nm (95% confidence interval: 345.5–394.5 nm; R^2^ = 0.97). (b) Western blot analysis of STAT6 phosphorylation at Tyr641. TF‐1 cells were treated with varying concentrations of D‐18252‐evo in the presence or absence of 1 ng/mL L‐IL‐4. Pascolizumab (10 nm) was included as a positive control. The immunoblot was independently repeated twice (*n* = 2 independent experiments) with consistent results.

In a cell‐based assay, D‐18252‐evo potently inhibited L‐IL‐4‐induced proliferation of TF‐1 cells. Cells were treated with increasing concentrations of D‐18252‐evo (0–800 nm) in the presence of 1 ng/mL L‐IL‐4 (Figure 7a). The compound exhibited a robust, dose‐dependent inhibitory effect, with an IC_50_ of 370 nm, indicating effective functional blockade of the IL‐4 signaling pathway. Western blot analysis confirmed that D‐18252‐evo dose‐dependently suppressed L‐IL‐4‐induced STAT6 phosphorylation (Figure 7b), with inhibitory efficacy comparable to pascolizumab, a well‐characterized IL‐4‐binding antibody. As a control, cells were analyzed in the absence of L‐IL‐4 stimulation to establish baseline phosphorylation levels. These results demonstrate that a functional D‐protein inhibitor targeting the L‐IL‐4‐receptor interface can be successfully developed using a combined de novo design and directed evolution approach.

## 3 Discussion

The landscape of mirror‐image protein engineering and design has evolved significantly beyond classical mirror‐image phage display. Mirror‐image binder discovery has been extended to monobody‐based platforms, including TRAP display against MCP‐1 and phage/yeast‐display workflows targeting the BCR::ABL1 SH2 domain. These approaches remain largely stochastic. They typically identify binding epitopes post hoc, offering limited control over the specific interface targeted. In contrast, our study establishes a rational, epitope‐driven workflow that integrates de novo computational design with a directed evolution strategy. A pivotal innovation in this work is the incorporation of WALTZ‐guided aggregation prediction as a filter for the directed evolution step. This approach allows for the simultaneous optimization of binding affinity and protein solution behavior, effectively deprioritizing variants prone to aggregation—a common pitfall in affinity‐centric maturation strategies.

Screening‐based mirror‐image discovery strategies typically require a chemically synthesized D‐enantiomer of the target as the selection bait; consequently, access to folded synthetic D‐targets is a practical prerequisite common to most mirror‐image pipelines, rather than a limitation unique to design‐based workflows. Building upon foundational ligation chemistries such as NCL [45], recent advances in chemical protein synthesis—including hydrazide‐enabled ligation, removable‐backbone and temporary‐support strategies, and modular NCL schemes—have significantly expanded the scope of synthetically accessible protein targets [44, 52, 53, 54, 55]. Post‐translational modifications (PTMs) present an additional consideration: while bioactive, non‐glycosylated recombinant forms of secreted proteins (including certain cytokines) are often available [56, 57, 58], mirror‐image glycosylation remains a significant challenge for targets requiring native glycans [59]. Moreover, several classes of PTMs can be introduced chemically, particularly those involving achiral functional groups; examples include methylation and acetylation [60, 61]. Importantly, these constraints apply broadly to all mirror‐image approaches reliant on synthetic D‐protein baits, regardless of whether binders are obtained through stochastic screening or structure‐guided design.

Within this scope, we developed a mirror‐image D‐protein inhibitor targeting human IL‐4. After optimizations of its properties by directed evolution, the engineered inhibitor, D‐18252‐evo, exhibits ∼90 nm binding affinity, good thermostability (maintaining structural integrity at 95°C), and robust resistance to proteolytic degradation (remaining intact after 20 h of pepsin exposure). These findings highlight the therapeutic potential of D‐proteins targeting IL‐4.

The thermal and proteolytic stability of D‐18252‐evo establishes a foundation for the development of novel biopharmaceuticals. Compared to conventional L‐protein therapeutics, D‐proteins display significantly enhanced in vivo stability, as evidenced by prior studies [29], offering distinct advantages for the long‐term management of chronic diseases such as asthma and allergic disorders. Moreover, their resistance to enzymatic degradation may present new opportunities for the development of orally bioavailable protein‐based therapeutics.

Our computational framework can be adapted to generate D‐protein inhibitors against other disease‐relevant targets. Future efforts should focus on optimizing the delivery strategies and pharmacokinetic properties of D‐18252‐evo, as well as evaluating its therapeutic efficacy in preclinical animal models. These steps will advance the clinical translation of D‐protein‐based modulators of IL‐4 signaling, paving the way for innovative treatments for immune‐mediated diseases. Finally, while ethical concerns and debates regarding hypothetical self‐replicating “mirror‐life” organisms have been raised [62, 63], our work is strictly confined to acellular, chemically synthesized D‐proteins used as therapeutics. These molecules lack nucleic acids and any capacity for replication, posing no risk of generating autonomous mirror organisms, thus aligning with current biosafety guidelines for mirror‐image molecular biology.

## 4 Materials and Methods

### 4.1 Computational Design

The crystal structure of the IL‐4–IL‐4Rα–IL‐13Rα1 complex (PDB ID: 3BPN) [7] was selected as the initial template and refined using PHENIX software [64]. The refined structure of L‐IL‐4 was used in subsequent steps. A theoretical model of D‐IL‐4 was generated by mirror‐imaging the coordinates of L‐IL‐4 across the y–z plane. Surface residues 29, 30, 32, 33, 36, 37, 40, 43, 44, 98, 99, 101, 102, 105, 106, 109, 112, 113, and 117 of D‐IL‐4 were identified as key interfacial residues (RIF residues) for downstream applications.

A rotamer interaction field (RIF) was generated for the RIF residues of the D‐IL‐4 target using RifGen. Initial docking of mini‐protein scaffolds to D‐IL‐4 was performed using PatchDock [65] to rapidly identify potential binding poses. These candidate models were further refined with the RifDock module [66], resulting in 6266 docked conformations.

The Rosetta FastDesign protocol [67] was applied to optimize the protein‐protein interfaces of the docked complexes, employing an energy function specifically adapted for heterochiral protein–protein interactions. Representative heterochiral complexes were selected as templates for constructing machine learning models. These models were iteratively refined using composite scoring functions and interfacial features to identify top‐ranking heterochiral complexes with optimal energetic and structural properties.

To enhance structural diversity, MotifGraft was utilized to extract key interaction motifs. A total of 441 D‐IL‐4‐interacting motifs were identified and grafted onto mini‐protein scaffolds, generating 3,141,719 design candidates. The heterochiral interfaces of these grafted models were subsequently optimized using the Rosetta FastDesign protocol. Ultimately, 7,888 D‐IL‐4 binders, each comprising 50–65 amino acids, were selected, and the corresponding oligonucleotide library was synthesized (Agilent).

### 4.2 Chemical Synthesis of D‐IL‐4 and D‐18252‐evo

#### 4.2.1 Peptide Segment Synthesis [68]

All peptides were synthesized via Fmoc‐based solid‐phase peptide synthesis (Fmoc SPPS) using a Liberty Blue microwave peptide synthesizer (CEM Corporation). Hydrazide‐terminated peptide fragments were assembled on a 2‐chlorotrityl chloride‐functionalized polystyrene‐divinylbenzene copolymer resin (1% DVB, 100–200 mesh, 0.43 mmol/g; Tianjin Nankai Hecheng Science & Technology Co., Ltd.), onto which hydrazine was added to form the hydrazide linker. In contrast, amide‐terminated fragments were synthesized on Rink Amide‐functionalized polystyrene‐divinylbenzene copolymer resin (1% DVB, 100–200 mesh, 0.38 mmol/g; Tianjin Nankai Hecheng Science & Technology Co., Ltd.), where the Rink Amide moiety served as the functional linker.

Fmoc deprotection was carried out at 90°C for 1 min with a solution of 10% piperidine and 0.1 M Oxyma in DMF, followed by three DMF washes. For amino acid coupling, the resin (0.25 mmol) was treated with four equivalents each of Fmoc‐protected amino acid (0.2 M, 5 mL in DMF), Oxyma (1 M, 1 mL in DMF), and DIC (0.5 M, 2 mL in DMF), and the reaction mixture was irradiated under microwave heating at 90°C for 2 min. The synthesis cycle concluded with three additional washes with DMF.

Following chain assembly, peptides were cleaved from the resin using a TFA‐based cleavage cocktail (TFA/TIPS/thioanisole/water/EDT, 82.5:5:5:5:2.5, v/v/v/v/v) for 3 h. The TFA solution was concentrated under a stream of nitrogen, precipitated with ice‐cold diethyl ether, centrifuged, and the supernatant was decanted. The crude peptide pellet was subsequently purified by preparative reversed‐phase high‐performance liquid chromatography (RP‐HPLC) to obtain the target peptide in high purity.

#### 4.2.2 Hydrazide‐Based Native Chemical Ligation [69]

The hydrazide peptide segment (1 mm) was dissolved in 6 M (Gd·Cl, pH 2.5). After incubation in an ice‐salt bath at −15°C, sodium nitrite (10 equivalents, 1 M) was added, and the oxidation reaction was allowed to proceed for 25 min. Subsequently, 4‐mercaptophenylacetic acid (MPAA, 50 equivalents) and an N‐terminal cysteine‐containing peptide segment (1.3 equivalents) were introduced. The pH was adjusted to 6.3–6.5 using a glass electrode, and the ligation mixture was stirred at room temperature overnight. The resulting ligation products were analyzed and purified by reversed‐phase HPLC.

#### 4.2.3 MPAA Thioester Peptide Synthesis [70]

Dissolve the crude hydrazide peptide segment (10 mg/mL) in 6 M Gd·Cl (pH 2.3). Add 10 equivalents of MPAA and 5 equivalents of pentanedione. Adjust the pH to 2.5 using a glass electrode. Stir the reaction mixture at room temperature for 2 h, then purify the product by reversed‐phase HPLC.

#### 4.2.4 Synthetic Route of D‐IL‐4

The mirror‐image configuration of human IL‐4 (D‐IL‐4) containing 129 residues was chemically synthesized. D‐IL‐4 was divided into three peptide segments to facilitate synthesis. Cys46 was introduced as 1,3‐thiazolidine‐4‐carbonyl (Thz), to avoid undesired cyclization. All three peptide segments were obtained by standard Fmoc SPPS, and the N‐terminus of peptide **1** was labeled with D‐biotin to enable subsequent BLI binding assays. Peptide **1** was purified by reversed‐phase high‐performance liquid chromatography (RP‐HPLC) on a C4 column (4.6 × 250 mm) using a linear gradient of 30‐80% buffer A in buffer B over 30 min, affording an isolated yield of 16%. Peptides **2** and **3** were purified under identical chromatographic conditions with a 20%–70% gradient, yielding 27% and 39%, respectively. Peptide **2** (25.9 mg, 1.0 equiv) and peptide **3** (16.8 mg, 1.2 equiv) were dissolved in 6 M Gd·Cl at pH 7.0. The pH was adjusted to 6.5 using a glass electrode. After 14 h of incubation, methoxyamine hydrochloride (MeONH_2_·HCl, 52 mg) was added to convert the Thz46 to Cys46. Tris(2‐carboxyethyl)phosphine hydrochloride (TCEP·HCl) was subsequently introduced until the pH reached 4.0. Following a 3‐h reduction period, the resulting product (peptide **4**) was purified by RP‐HPLC on a C4 column (10 × 250 mm, 300 Å) with a 20%–70% gradient over 30 min, yielding 42% of the desired product. Native chemical ligation between peptide **1** (7.4 mg) and peptide **4** (16.1 mg) was performed under standard conditions. The final product (peptide **5**) was purified using the same chromatographic setup as for peptide **4**, but with a 30‐80% gradient, resulting in a 36% isolated yield. The identity of peptide **5** was confirmed by electrospray ionization mass spectrometry (ESI‐MS). The overall yield for the multi‐step synthesis was 15% (Figure S1).

#### 4.2.5 Synthetic Route of D‐18252

D‐18252 was synthesized via stepwise Fmoc‐based solid‐phase peptide synthesis. The crude product was purified by reversed‐phase HPLC using a C4 column (4.6 × 250 mm) with a linear gradient of 20%–70% solvent A in solvent B over 30 min. The isolated yield after RP‐HPLC purification was 7% (Figure S7).

#### 4.2.6 Synthetic Route of D‐18252‐evo

D‐18252‐evo was synthesized using stepwise Fmoc‐based solid‐phase peptide synthesis. The crude product was purified by reversed‐phase HPLC on a C4 column (4.6 mm × 250 mm) with a linear gradient of 20%–70% solvent A in solvent B over 30 min. The isolated yield after purification was 12% (Figure S9).

### 4.3 Protein Purification

#### 4.3.1 Expression, Purification, and Folding of L‐IL‐4

The coding sequence of human IL‐4 was cloned into the pET‐28b vector with an N‐terminal 6×His tag. Following confirmation by colony PCR and DNA sequencing, the recombinant plasmid was transformed into competent Lemo21(DE3) cells. Transformed *E. coli* were grown in LB medium at 37°C until the OD_600_ reached 0.8, and protein expression was induced with 1 mM IPTG (final concentration) for 3 h at 37°C. Induced cells were harvested by centrifugation at 10 000 × g, resuspended in PBS (pH 7.4), and lysed by sonication. The lysate was centrifuged to isolate the inclusion bodies, which were solubilized in buffer containing 0.05 M Tris‐HCl (pH 8.0) and 6 M Gd·Cl, followed by centrifugation (12 000 × g, 30 min) to remove insoluble debris. The supernatant was loaded onto a gravity‐flow column packed with 1.5 mL Ni Sepharose 6 Fast Flow resin (Cytiva Life Sciences) and eluted with 0.05 M Tris‐HCl (pH 8.0), 6 M Gd·Cl, and 300 mM imidazole. The eluted protein was treated with 10 mm DTT for 30 min at room temperature to fully reduce disulfide bonds. The solution was then diluted to 0.1 mg/mL in folding buffer (0.05 M Tris‐HCl, pH 8.0, 6 M Gd·Cl) containing 2 mm reduced glutathione and 0.2 mm oxidized glutathione. The diluted protein was dialyzed overnight at 4°C against 20 mm Tris‐HCl (pH 8.0) and 150 mm NaCl. The folded protein was concentrated and further purified by SEC using an ÄKTA pure system (Cytiva Life Sciences). D‐IL‐4 was refolded using the identical protocol.

#### 4.3.2 Expression and Purification of L‐18252 and Its Mutants

The coding sequence of L‐18252 was cloned into the pET‐28b vector to include an N‐terminal 6×His tag. The resulting plasmid was transformed into Lemo21(DE3) competent cells. Transformed *E. coli* cultures were grown in LB medium at 37°C until the OD_600_ reached 0.6, after which protein expression was induced with 0.1 mm IPTG (final concentration) and cultures were incubated at 18°C overnight. Cells were harvested by centrifugation (10 000 × g, 10 min), resuspended in lysis buffer (20 mm Tris, pH 8.0, 500 mm NaCl), and lysed by sonication. The lysate was clarified by centrifugation (20 000 × g, 1 h), and the supernatant was loaded onto a gravity column containing 1.5 mL of Ni Sepharose 6 Fast Flow resin (Cytiva Life Sciences) for affinity chromatography. Further purification was performed by SEC using an ÄKTA pure system equipped with a Superdex 75 Increase 10/300 GL column (Cytiva Life Sciences), with PBS (pH 7.4) as the mobile phase. The purified protein was analyzed by SDS‐PAGE, and fractions containing the target protein were pooled, concentrated, flash‐frozen in liquid nitrogen, and stored at −80°C. The same expression and purification protocol was applied to all other mutants.

### 4.4 Biochemical and Biophysical Characterization

#### 4.4.1 Circular Dichroism

Far‐UV CD spectra were recorded using a Chirascan V100 spectropolarimeter (Applied Photophysics). Scans were performed over the wavelength range of 180–260 nm at 25°C and 95°C, with a subsequent measurement at 25°C to evaluate structural reversibility. For thermal denaturation experiments, the CD signal at 222 nm was monitored with a heating rate of 2°C/min and an equilibration time of 30 s per temperature increment.

#### 4.4.2 Biolayer Interferometry

Binding affinities between target proteins and their binders were determined using an Octet RED96e system (ForteBio). Biotinylated D/L‐IL‐4 was immobilized onto streptavidin‐coated biosensors (SA, Sartorius). After baseline stabilization, the biosensors were exposed to solutions containing either L‐18252‐evo or D‐18252‐evo to monitor the association phase, followed by dissociation in phosphate‐buffered saline (PBS, pH 7.4) supplemented with 0.02% Tween 20. Binding curves generated at varying analyte concentrations were analyzed using Octet Data Analysis HT software and a steady‐state model to determine binding affinities.

#### 4.4.3 Size‐Exclusion Chromatography Co‐Migration Assay

The interaction between L‐IL‐4 and D‐18252‐evo was assessed using SEC. L‐IL‐4, D‐18252‐evo, and a mixture of both were separately loaded onto a Superdex 75 Increase 10/300 column (GE Healthcare). Eluted fractions were collected and analyzed by SDS‐PAGE to confirm co‐elution, indicative of complex formation.

#### 4.4.4 Protease Stability Assay

Digestion of L‐18252‐evo and D‐18252‐evo (0.2 mg/mL each) was carried out at 37°C using either trypsin (Genom, 2.2 mg/mL) in Hank's Balanced Salt Solution (HBSS) containing 0.02% EDTA or pepsin (Aladin, 0.22 mg/mL) in 0.1 M glycine buffer (pH 2.5).

### 4.5 Yeast Display

#### 4.5.1 DNA Library Preparation

DNA sequences encoding the designed proteins were codon‐optimized for expression in *Saccharomyces cerevisiae* using DNAWorks 2.0 [71]. An oligonucleotide pool was synthesized by Agilent Technologies [72]. The pool was amplified by PCR using KOD polymerase, and the resulting amplicons were separated on a 1.5% agarose gel and purified with the TIANGEN Gel Extraction Kit. The pETCON vector backbone was linearized via PCR, and the library DNA was co‐transformed with the linearized vector into *S. cerevisiae* EBY100 cells. Successful library transformation was confirmed by obtaining more than 1 × 10^7^ transformants. Transformed yeast cells were cultured in 50 mL of SDCAA medium at 30°C overnight until the OD_600_ exceeded 10, then mixed with an equal volume of 50% (v/v) glycerol and stored at −80°C.

#### 4.5.2 Yeast Display and Screening

Following an established protocol [73], yeast cells cultured in SDCAA medium (OD_600_ > 2) were harvested by centrifugation, resuspended in SGCAA medium, and induced at 20°C for 20–28 h. A total of 1 × 10^8^ yeast cells were incubated at room temperature for 30 min with anti‐c‐Myc FITC (1:250, Miltenyi Biotech) and biotinylated D‐IL‐4, followed by an additional 30‐min incubation with streptavidin‐phycoerythrin (SAPE, 1:100, R&D Systems). Cells were washed twice with phosphate‐buffered saline containing 1% bovine serum albumin (PBSF), resuspended in PBS, and double‐positive cells were isolated using a BD Melody cell sorter. Four consecutive rounds of screening were conducted with D‐IL‐4 concentrations of 1 µm, 100 nm, 10 nm, and 1 nm. In each round, the top 1 × 10^4^ double‐positive cells were collected, transferred to 5 mL of SDCAA medium, and cultured overnight at 30°C. An equal volume of 50% (v/v) glycerol was added, and the cultures were stored at −80°C.

After screening, plasmids were extracted from yeast cells using the TIANprep Yeast Plasmid DNA Kit and used as templates for 100 µL PCR amplifications (30 cycles) to incorporate round‐specific barcodes. PCR products were purified by gel electrophoresis, and DNA fragments from all rounds were pooled and subjected to high‐throughput sequencing (Novogene).

#### 4.5.3 Combinatorial Library Construction

Based on SSM and structural analysis, combinatorial mutations were introduced at the binding interface using degenerate codon‐designed primers in a PCR amplification. The resulting PCR products were purified and co‐electroporated with a linearized pETCON vector into yeast cells. After overnight culture, transformants were plated and sequenced to evaluate library quality.

#### 4.5.4 Error‐prone PCR Library Construction

Random mutagenesis was performed using GeneMorph II kits (Agilent) in 40 µL reaction volumes containing 0.05 ng of template DNA, with 40 amplification cycles to yield an average of approximately three amino acid substitutions per sequence. The resulting mutagenized library was co‐transformed with linearized vector fragments into *S. cerevisiae* EBY100 cells, followed by colony picking and sequencing to confirm mutation frequencies.

### 4.6 Functional Validation of D‐18252‐evo

#### 4.6.1 TF‐1 Cell Proliferation Assay [74]

TF‐1 cells were cultured in RPMI‐1640 medium supplemented with 2% fetal bovine serum and exposed to varying concentrations of D‐18252‐evo in the presence of IL‐4 for 48 h under standard conditions (37°C, 5% CO_2_). Cellular ATP levels were quantified using the ApoSENSOR Cell Viability Assay Kit (BioVision), and luminescence was measured with a Thermo Varioskan LUX microplate reader. Data were analyzed using Prism 10 (GraphPad Software).

#### 4.6.2 Detection of STAT6 Phosphorylation

STAT6 phosphorylation levels were assessed to evaluate the effect of D‐18252‐evo on IL‐4‐induced STAT6 activation [75]. After a 4‐h serum starvation period, TF‐1 cells were stimulated with 1 ng/mL IL‐4 and simultaneously treated with increasing concentrations of the compound. Cells were incubated at 37°C for 15 min, followed by lysis. Whole‐cell lysates were analyzed by western blotting using antibodies against total STAT6 and phosphorylated STAT6 (Tyr641) (Cell Signaling Technology).

### 4.7 Statistical Analysis

All statistical analyses were performed using GraphPad Prism (version 10). For cell proliferation assays, dose–response curves were fitted using the “log(inhibitor) versus normalized response – variable slope” model to estimate IC_50_. Within each independent experiment, each concentration was measured in duplicate (technical replicates). Data are presented as the mean of duplicate wells, with error bars indicating the standard deviation (SD) of the technical replicates. The experiment was repeated independently twice (*n* = 2 independent experiments), yielding consistent dose–response trends and similar IC_50_ values. Percent proliferation was normalized on each plate by defining 0% as the signal from wells without L‐IL‐4 stimulation and 100% as the signal from wells stimulated with L‐IL‐4 alone. This analysis yielded an IC_50_ of 369.9 nm (95% confidence interval: 345.5–394.5 nm) and a high goodness‐of‐fit (R^2^ = 0.9741). The reported 95% confidence interval for the IC_50_ was obtained from the nonlinear regression fit and does not account for variability between experiments. Inhibitor concentrations were plotted on a log_10_ scale. For CD experiments, spectra were recorded from 180 to 260 nm, and each spectrum represents the average of two consecutive scans of the same sample (*n* = 2 technical replicates). Duplicate scans were baseline‐corrected and averaged for subsequent analysis. BLI data were analyzed using Octet Data Analysis software, and the K_D_ values were determined by fitting the data to a steady‐state 1:1 binding model across a concentration series. Detailed fitting parameters—including K_D_ (M), K_D_ error, R_max, R_max error, Chi^2^, R^2^ and the fitting model—are provided in Table S1. All BLI fits exhibited high quality, with R^2^ values greater than 0.98. Unless otherwise specified, the reported “K_D_±error” reflects the fitting error derived from Octet Data Analysis. Representative results from protease stability and Western blot analyses are presented to illustrate protein stability and phosphorylation status, respectively. Both assays were independently repeated twice (*n* = 2). The statistical approach in this study was restricted to parameter estimation (e.g., IC_50_, K_D_) and assessment of data quality (e.g., R^2^ values).

### 4.8 Ethics Statement

All experiments were conducted using isolated, chemically synthesized proteins and well‐established cultured cell lines, in accordance with institutional biosafety regulations and standard laboratory protocols. The study does not involve nucleic acids, replication‐competent systems, or any form of self‐replicating “mirror‐life” organisms.

## Author Contributions

K.S., L.L., and P.L. conceived and supervised the project. L.X. and Y.R. contributed equally to this work. K.S. developed the computational method and designed the D‐protein binders. L.X. and H.L. performed the refolding, yeast screening, and binder evaluation experiments. Y.R. and T.W. chemically synthesized the D‐IL‐4 and D‐protein binders. Y.Y. performed cell‐based assays. P.L. and L.X. wrote the original draft and all authors participated in manuscript revision.

## Conflicts of Interest

L.X., K.S., Y.R., L.L., and P.L. are inventors on a provisional patent application submitted by Westlake University, covering the function of D‐proteins described in this study.

## Supporting information




**Supporting File**: advs74962‐sup‐0001‐SuppMat.docx.

## Data Availability

The data that support the findings of this study are available from the corresponding author upon reasonable request.
