# Supplementary material for: De Novo Design and Directed Evolution Refinement of Mirror‐Image Protein Binders Targeting Interleukin‐4
Source: Adv Sci (Weinh). 2026 Mar 31;13(32):e15425. doi: 10.1002/advs.202515425 (PMC13252646; doi:10.1002/advs.202515425)
Supplement: Supplementary file 1 — Supporting File: advs74962‐sup‐0001‐SuppMat.docx. [file ADVS-13-e15425-s001.docx]

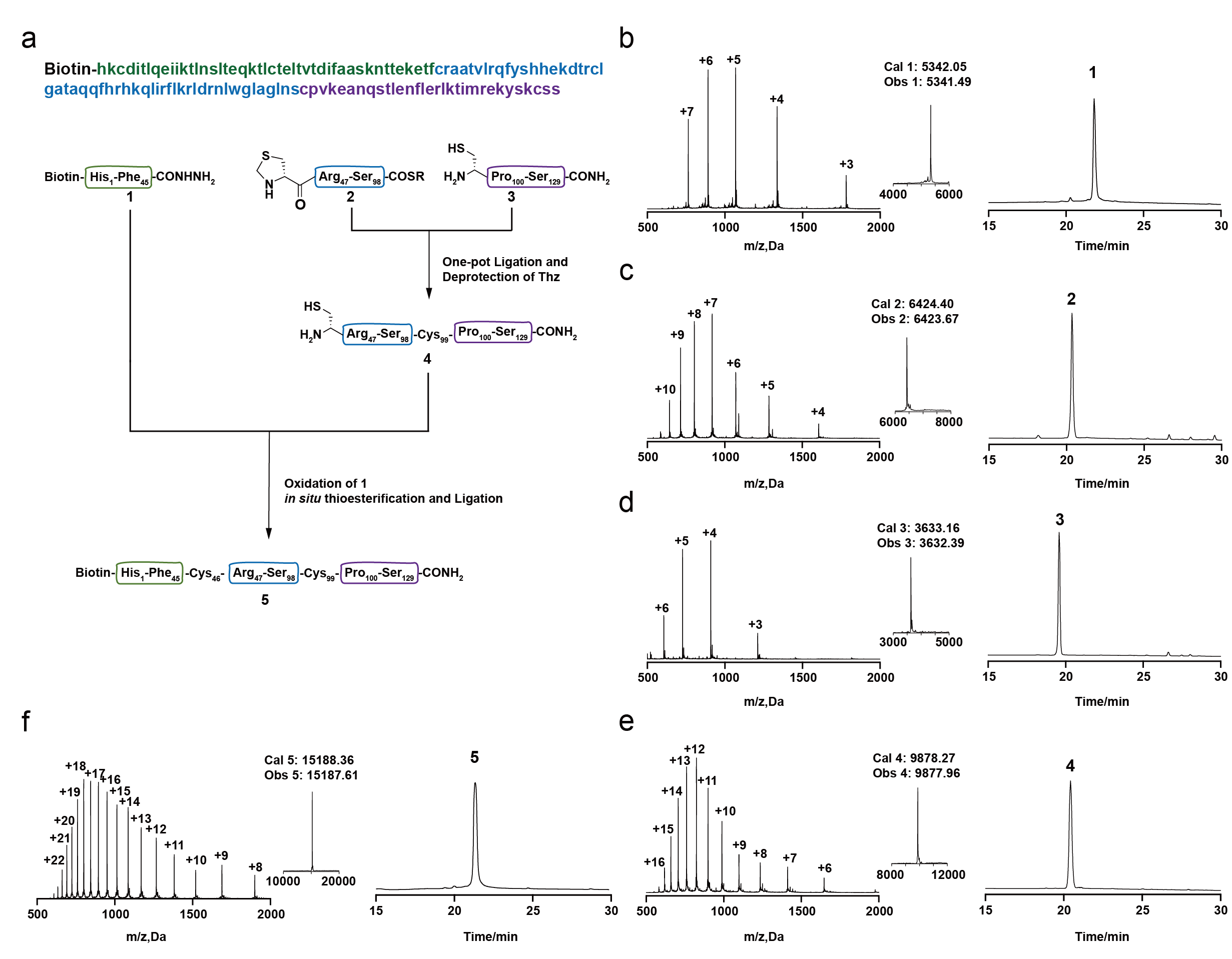


**Figure S1.** Synthetic route and characterization of D-IL-4. (a) Overall synthetic route of D-IL-4. (b-f) HPLC analysis and ESI-MS characterization of purified intermediates 1-5.


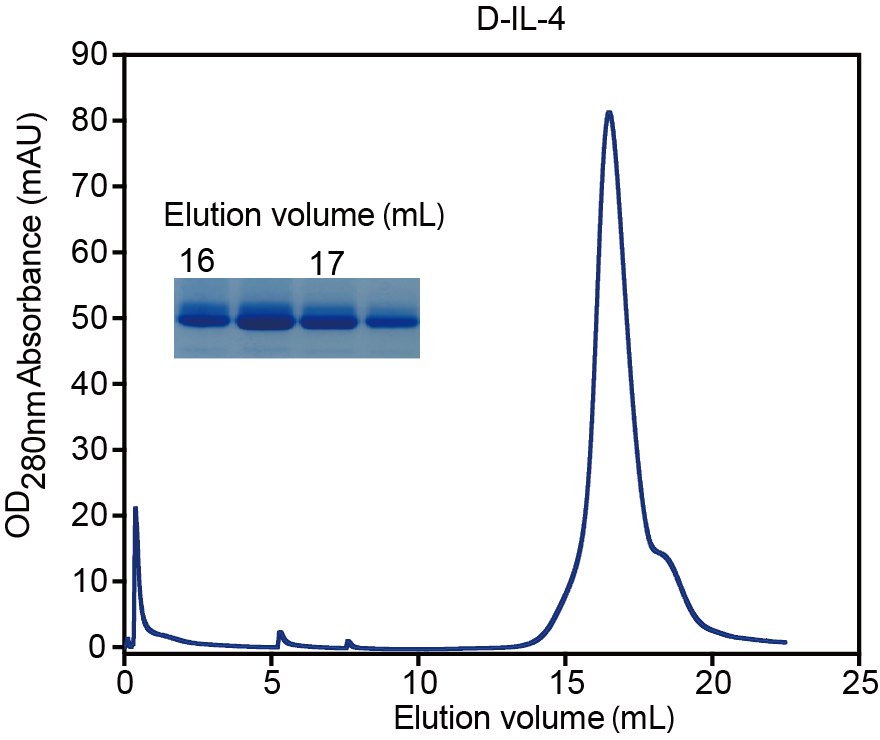


**Figure S2.** Gel filtration chromatography and SDS-PAGE analysis of folded D-IL-4.


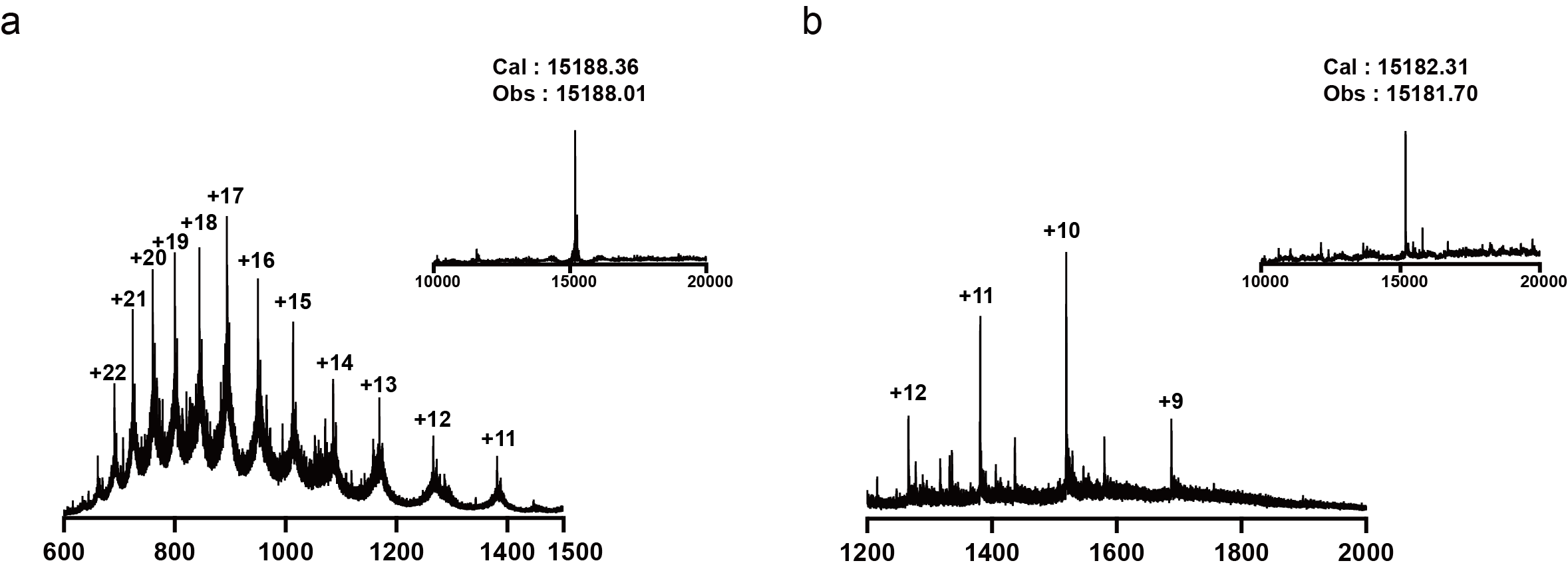


**Figure S3.** HRMS analysis of D-IL-4. (a) HRMS spectrum of unfolded D-IL-4, showing an observed mass of 15,188.01 Da. (b) HRMS spectrum of folded D-IL-4 after oxidative folding, showing an observed mass of 15,181.70 Da. The observed mass decrease is consistent with oxidation and formation of three disulfide bonds.

**
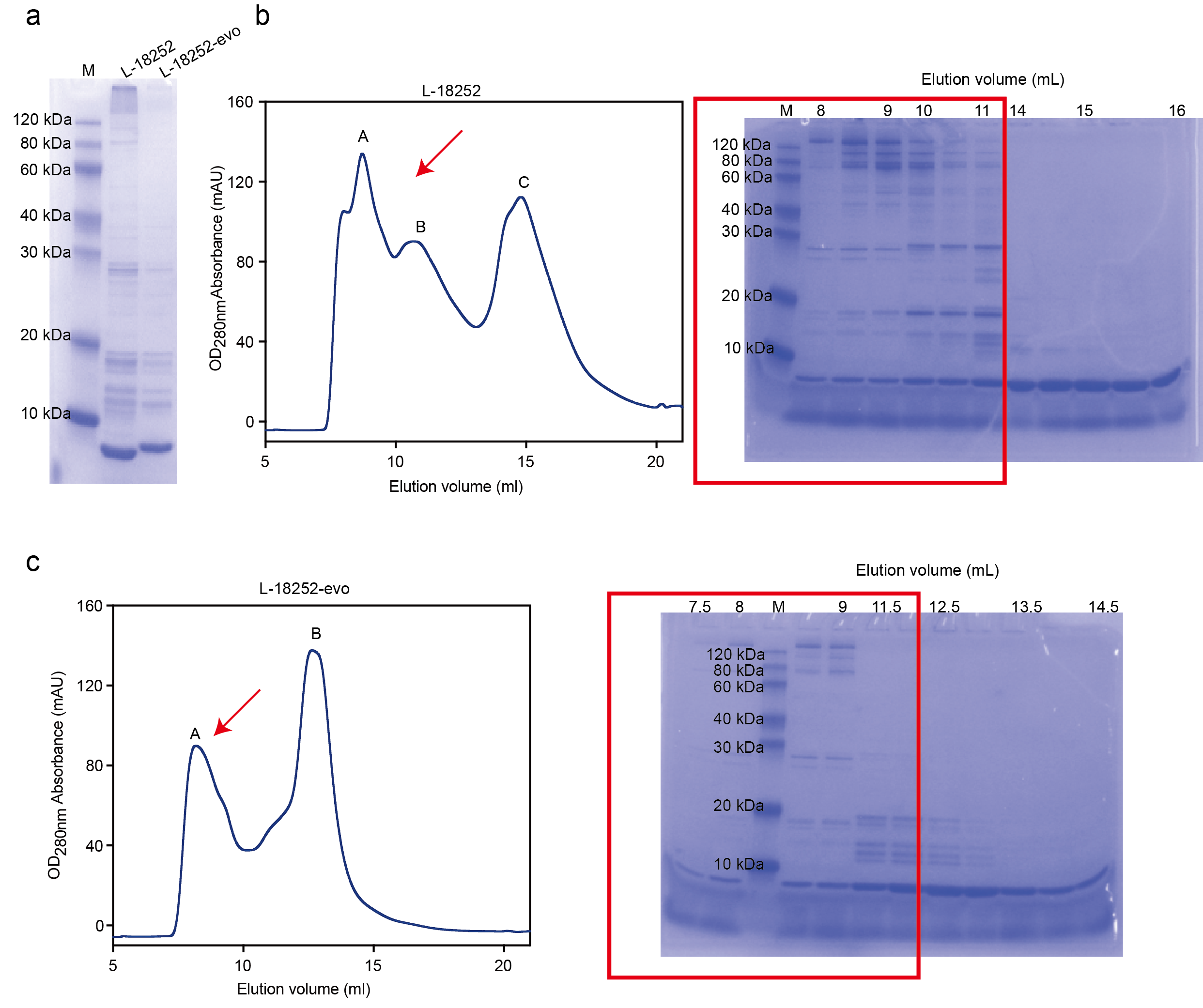
**

**Figure S4**. Comparison of L-18252 and L-18252-evo**. (a)** SDS-PAGE analysis of the Ni^2^⁺-affinity chromatograph eluates for L-18252 and L-18252-evo. **(b)** SEC and SDS-PAGE analysis of L-18252. (c) SEC and SDS-PAGE analysis of L-18252-evo. The arrow indicates the aggregate peak co-eluting with the host protein; the boxed region highlights the elution fractions analyzed by SDS-PAGE.


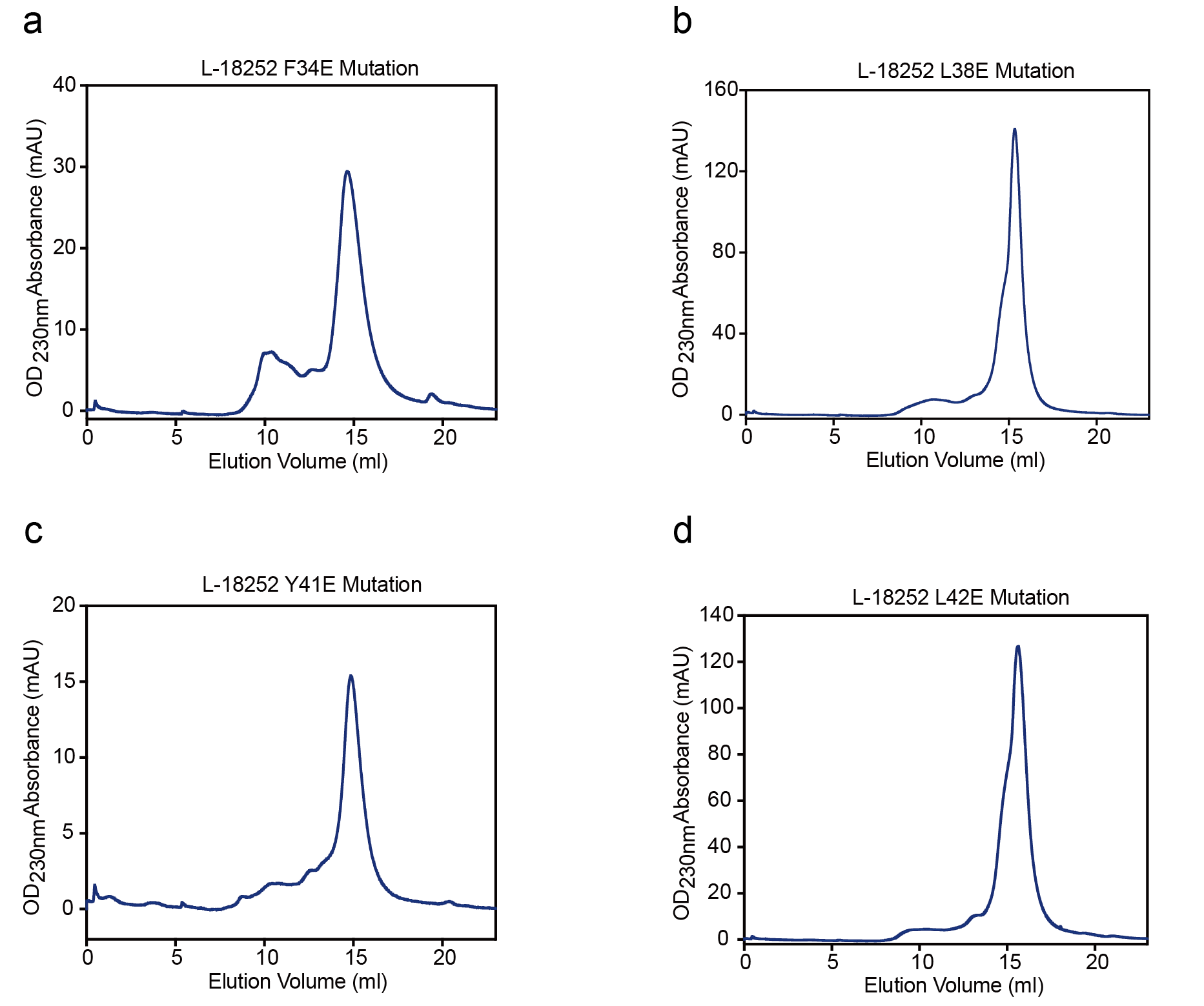


**Figure S5**. SEC profiles of L-18252 point mutants. (a) F34E, (b) L38E, (c) Y41E, and (d) L42E**. Proteins were purified to homogeneity by SEC on a Superdex 75 Increase 10/300 GL column, and the collected fractions were used directly in BLI binding assays with D-IL-4.**


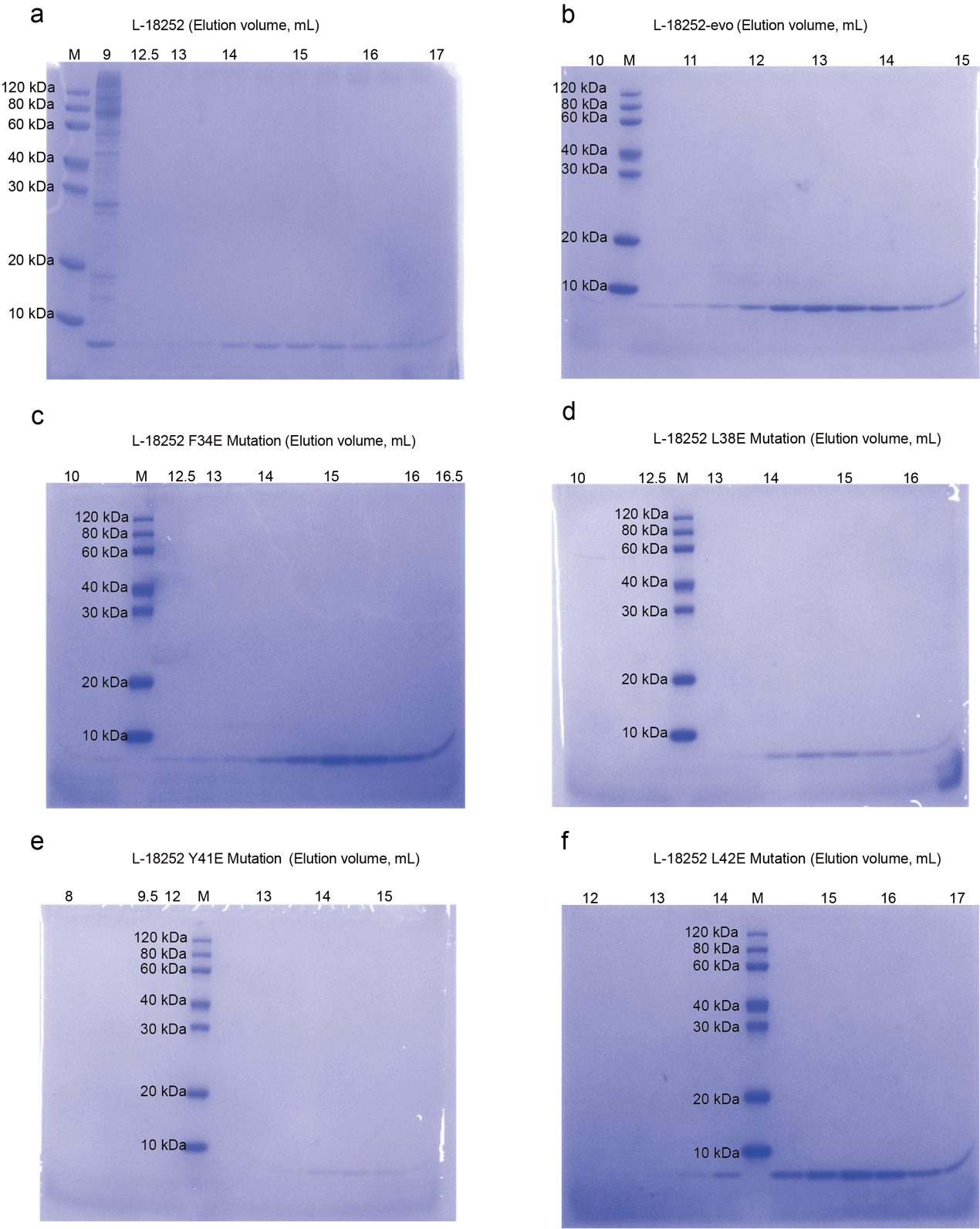


**Figure S6.** SDS-PAGE characterization of L-18252, L-18252-evo, and single-point mutants. Purified proteins were analyzed by SDS-PAGE, including L-18252 (a), L-18252-evo (b), and the L-18252 single-point mutants F34E (c), L38E (d), Y41E (e), and L42E (f). Elution volumes (in mL) for each lane are indicated above the gel. Molecular weight markers (in kDa) are shown on the left and correspond to 10, 20, 30, 40, 60, 80, and 120 kDa.
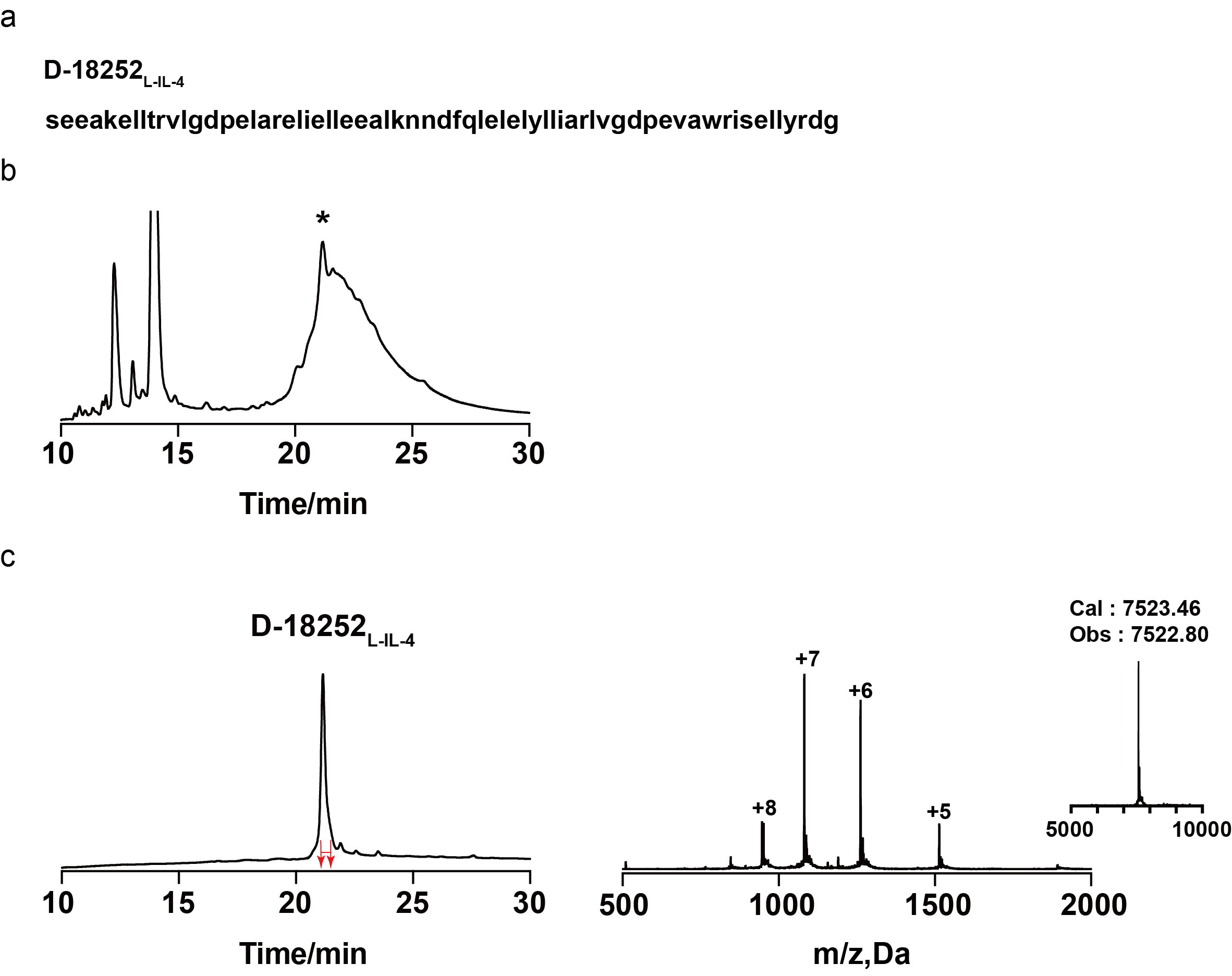


**Figure S7.** Sequence and characterization of D-18252. (a) Amino acid sequence of D-18252. (b) HPLC analysis of crude D-18252. (c) HPLC analysis and ESI-MS characterization of purified D-18252.

**
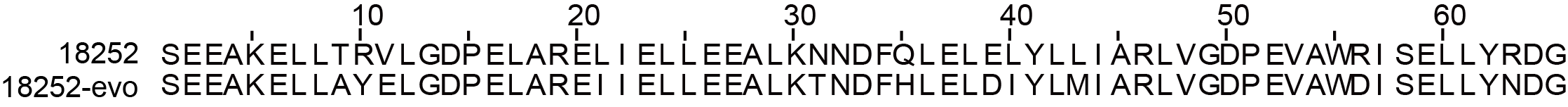
**

**Figure S8.** Sequence alignment of L-18252 and L-18252-evo. The mutations in L-18252-evo that differentiate it from L-18252 are located at residues 9, 10, 11, 21, 31, 35, 39, 40, 43, 56, and 63.


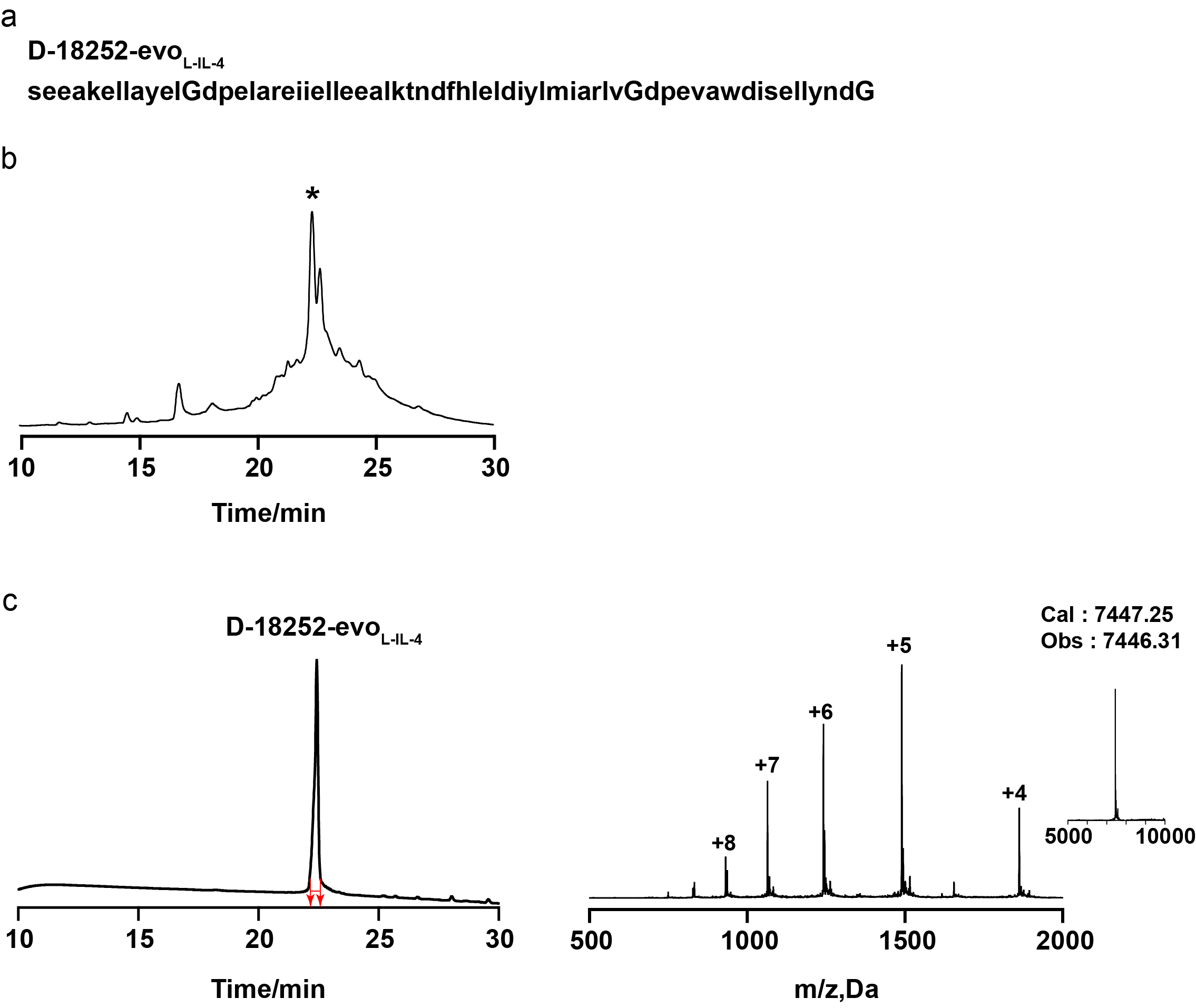


**Figure S9.** Sequence and characterization of D-18252-evo. (a) Amino acid sequence of D-18252-evo. (b) HPLC analysis of crude D-18252-evo. (c) HPLC chromatogram and ESI-MS spectrum of purified D-18252-evo.


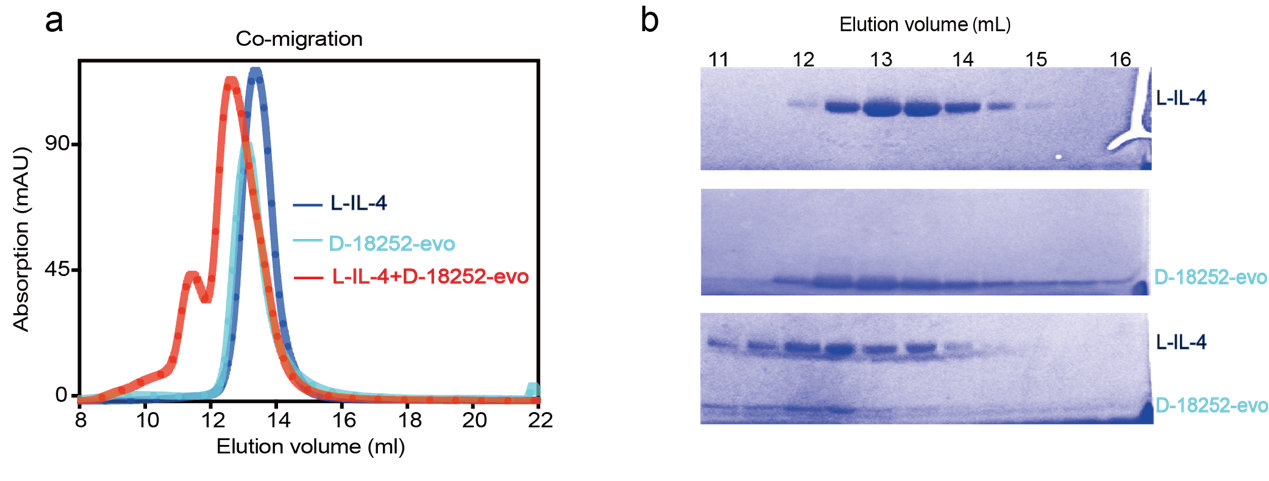


**Figure S10.** The D-18252-evo co-migrated with L-IL-4. (a) SEC analysis shows co-migration of D-18252-evo (light blue) and L-IL-4 (dark blue), with the resulting complex eluting earlier (red). (b) SDS-PAGE analysis (Coomassie staining) of SEC fractions (11-16 mL) confirms the presence of both components in the eluted complex.

|  | KD (M) | KD Error | Rmax Error | Rmax | Chi^2 | R^2 | Fitting Model |
| --- | --- | --- | --- | --- | --- | --- | --- |
| D-IL-4/L-18252 | 2.20E-07 | ± 2.6E-08 | ± 0.042 | 0.9975 | 0.0028 | 0.9895 | Steady-State |
| D-IL-4/L-18252-evo | 6.60E-08 | ± 9.0E-09 | ± 0.018 | 0.4231 | 0.0018 | 0.9809 | Steady-State |
| L-IL-4/D-18252 | 2.30E-06 | ± 2.7E-07 | ± 0.017 | 0.3149 | 0.0001 | 0.9939 | Steady-State |
| L-IL-4/D-18252-evo | 8.70E-08 | ± 1.3E-08 | ± 0.013 | 0.2616 | 0.0004 | 0.9815 | Steady-State |

**Table S1.** BLI data Fitting for IL-4.
